# The Neuroprotective Effects of Caffeine in a Neonatal Hypoxia-Ischemia Model are Regulated through the AMPK/mTOR Pathway

**DOI:** 10.7150/ijbs.101087

**Published:** 2025-01-01

**Authors:** Maria. E. Bernis, Hannah Burkard, Anna-Sophie Bremer, Kora Grzelak, Margit Zweyer, Elke Maes, Efe Nacarkucuk, Hanna Kaibel, Charlotte Hakvoort, Andreas Müller, Hemmen Sabir

**Affiliations:** 1 Department of Neonatology and Pediatric Intensive Care, Children's Hospital University of Bonn, Bonn, Germany.; 2 Deutsche Zentrum für Neurodegenerative Erkrankungen (DZNE), Bonn, Germany.

**Keywords:** Neonatal, Hypoxia-Ischemia, AMPK, mTOR, Neuroprotection, Caffeine

## Abstract

Neonatal hypoxic-ischemic encephalopathy (HIE) is the most common cause of death and long-term disabilities in term neonates. Caffeine exerts anti-inflammatory effects and has been used in neonatal intensive care units in recent decades. In our neonatal rat model of hypoxic-ischemic (HI) brain injury, we demonstrated that a single daily dose of caffeine (40 mg/kg) for 3 days post-HI reduced brain tissue loss and microgliosis compared to the vehicle group. The AMPK/mTOR pathway plays an important role in sensing the stress responses following brain injury. However, the role of mTOR in HI-associated brain damage remains unclear. A detailed analysis of the AMPK/mTOR pathway in our model revealed that this pathway plays a key role in hypoxia-regulated neuroprotection and can be significantly influenced by caffeine treatment. Targeting HI with caffeine might offer effective neuroprotection, reduce mortality, and improve functional outcomes in patients with HIE, especially in low- and middle-income countries, where neuroprotective treatment is urgently needed.

## Introduction

Neonatal hypoxic-ischemic encephalopathy (HIE) affects approximately 3-5 neonates per 1000 live births in high-income countries and 10-20 neonates per 1000 live births in low- and middle-income countries (LMIC) 1. Up to 40 % of neonates with perinatal asphyxia have moderate neonatal encephalopathy with a large spectrum of impairments in motor and cognitive functions, whereas severe encephalopathy often leads to death 2. Despite extensive research on various treatment strategies, therapeutic hypothermia (TH) remains the only approved and standard treatment for neonates with moderate to severe HIE 3. However, TH is only partially effective given that many infants still experience severe brain damage despite cooling treatment 4-6. Moreover, in LMICs TH has been shown to be non-neuroprotective, and this difference may be due to different underlying etiologies of encephalopathy, poor nutrition, and co-existence of infection and inflammation 5, 7. Further research is essential to test promising neuroprotective treatments that could save thousands of newborns in LMICs. During HIE, a reduction of cerebral blood flow and oxygen delivery results in energy depletion and inability to maintain cellular homeostasis 8. A promising approach in the search for new neuroprotective treatments will require better understanding of the key molecular signals that maintain homeostasis in the brain. The AMP-activated protein kinase (AMPK) and mammalian target of rapamycin (mTOR) pathways orchestrate opposing signaling pathways involved in sensing the availability of nutrients and energy and maintaining cellular homeostasis 9. From a functional perspective, mTOR is considered a molecular sensor of the cellular energy status 10. AMPK, a negative regulator of mTOR activity, senses the cellular energy status and glucose availability. A reduction in oxygen levels promotes ATP/AMP imbalance, inducing AMPK activation and mTOR inhibition 11. Recently, we demonstrated that caffeine is a potential candidate for preventing neonatal hypoxic-ischemic (HI) brain injury 12. Caffeine is a methylxanthine drug that has been used as a standard treatment in the neonatal intensive care units to treat neonatal apnea over the last 30 years 13. Caffeine is a promising candidate as a potential neuroprotective drug in LMICs, because its use in neonates is safe and accessible. It has been demonstrated that caffeine crosses the placenta and the blood-brain barrier and has been shown to have anti-oxidative, anti-inflammatory, and anti-apoptotic activities 14, 15. It has also been reported to inhibit kinase activity, including mTOR 16. However, the exact mechanism underlying the neuroprotective effects of caffeine in a neonatal hypoxia-ischemia model remains unknown. In the present study, we investigated the regulatory mechanism of AMPK/mTOR in a neonatal hypoxia-ischemia model as a potential target for the development of new treatments. We focused our research on the characterization of caffeine as a potential treatment and elucidated how caffeine affects the AMPK/mTOR pathway as a potential target for the neuroprotective effect observed after treatment in a neonatal hypoxia-ischemia model.

## Material and Methods

### Animals and experimental procedure

Experiments were performed as previously described 17-19, following the ARRIVE guidelines, and according to the Animal Protection Committee of the North Rhine-Westphalia State Environment Agency (LANUV), Germany. All studies were performed using 7-day-old (P7) Wistar rat pups of both sexes. Animals were kept at the Central Animal Laboratory of the Deutsches Zentrum für Neurodegenerative Erkrankungen (DZNE) Bonn, Germany, with a 12:12 h dark/light cycle at an environmental temperature of 21°C, with food and water *ad libitum*. All animals were randomized across litter, sex, and weight for all treatments before commencing experiments. The average litter size consisted of 10-12 pups. All experiments and analysis were performed by two observers blinded to the different treatments 20, 21. A total of 295 animals were used in the different treatment groups and sacrificed at different time points (see Supplementary Table 1).

Temperature was monitored in “sentinel” rat pups which were not from the treatment groups 17-19 using a rectal probe (IT-21, Physitemp Instruments, Clifton, NJ, USA) connected to a servo-controlled cooling machine and a cooling mat (CritiCool, MTRE, Yavne, Israel). The sentinel pup maintained the nesting temperature of P7 rat pups 22 or the treatment temperatures during the experiments (see below). The standardized HI Vannucci rat model was used as previously described 17-19. Briefly, the left common carotid artery was ligated and cut under general isoflurane anesthesia following 90 min of hypoxia (8 % O_2_) at a rectal temperature (T_rectal_) of 36°C, resulting in moderate HI brain injury 20, 21. Immediately after HI, all animals were treated with normothermia (NT) at T_rectal_ of 37 °C for 5 h, as previously described 20, 21, 23. Sham animals were maintained under general isoflurane anesthesia for 5 min. The pups were returned to their dams following the treatment period and were sacrificed using a single intraperitoneal injection of Chloralhydrate (450 mg/kg) at different time points (4 h after HI, 24 h after HI, 7-days after HI, and 60-days after HI). All animals gained weight over the days of experiments. The median weight was 11.5 g at the day of the surgery and 38.5 g seven-days after HI. At the later time points (7 or 60-days after HI), the animals were sacrificed by transcardiac perfusion fixation with 10 % neutral-buffered formalin, and their brains were stored in 10 % neutral-buffered formalin until further processing. Coronal blocks were cut (3 mm sections), and embedded in paraffin. Ten-micrometer slices were cut from the two neighboring blocks representing the cortex, hippocampus, basal ganglia, and thalamus (distance to bregma, -3.8 mm). Staining with hematoxylin and eosin (HE) were performed, and the slices were scanned (Epson Perfection V750 Pro). The hemispheric areas were analyzed using ImageJ software. The ipsilateral side was compared to the contralateral side, and the area loss of the ligated side was calculated using the formula (1 - (area left/area right) × 100), as previously described 18, 21, 22.

### AMPK/mTOR inhibitor or caffeine treatment

All injected drugs were administered intraperitoneally (i.p.). Each animal received a final dose of 0.1 ml/10 g body weight.

• Caffeine citrate was prepared in saline to obtain the final concentrations used (15, 20, 40, and 120 mg/kg) 12. For all concentrations, the first dose was administered before hypoxia, with two subsequent doses at 24 and 48 h after HI. We also administered a first dose of 40 mg/kg after hypoxia, following two doses at 24 and 48 h after HI. Sham groups treated with caffeine received three single dose of caffeine (40 mg/kg) at P7, P8 and P9.

• Compound C is a potent and reversible AMPK inhibitor that competes with ATP in the absence of AMP and inhibits AMPK phosphorylation 24. Compound C (Sigma - Germany) was prepared in saline solution at a final concentration of 5 mg/kg. A single dose was administered to the animals before hypoxia. Due to the high mortality rate, a second dose was not administered following hypoxia.

• Rapamycin is an inhibitor of mTOR which binds to the hydrophobic binding pocket of the cytosolic protein FKBP12, thereby inhibiting the activation of 4EBP1 and S6 25. Rapamycin (Tocris - UK) was prepared in saline to a final concentration of 10 mg/kg. The first dose was administered before hypoxia, followed by the second dose immediately after hypoxia.

### Pharmacokinetics following caffeine determination

Pharmacokinetic study was performed in the University of Michigan. To determine caffeine in rat serum or plasma, 160 μL of internal standard solution (Caffeine-d3 (purity >98.0%, Cayman Chemical (MI, USA)) and 50 ng/mL in acetonitrile (LC-MS Grade-Fischer Chemical (MI, USA)) and 30 μL of acetonitrile were added to 30 μL of serum or plasma samples. The mixture was vortexed for 10 min and centrifuged at 3500 rpm for 10 min. The supernatant was then transferred to autosampler vials for LC-MS/MS analysis. Brain samples (Contralateral or Ipsilateral) were homogenized (Precellys tissue homogenizer, Bertin Technologies, Montigny-le-Bretonneux, France) with the addition of a 20% acetonitrile-water solution at a ratio of 5:1 volume (mL) to weight of tissue (g). Then, 160 μL of the internal standard solution and 30 μL of acetonitrile were added to 30 μL of tissue homogenization samples for protein precipitation. The mixture was vortexed for 10 min and centrifuged at 3500 rpm for 10 min. The supernatant was then transferred to autosampler vials for LC-MS/MS analysis.

Blank serum, plasma and brain samples from untreated control groups were used to exclude contamination or interference. The Caffeine analytical curves were constructed with 12 nonzero standards spiked in blank serum, plasma, or brains by plotting the peak area ratio of caffeine to the internal standard versus the sample concentration. The concentration range evaluated ranged from 1 to 5000 ng/mL in the rat serum, plasma, and brain. Quality control samples were prepared from separate weighed powders at concentrations of 5, 2000, 5000 ng/mL. Quality control samples were run before, during, and after the samples to evaluate the accuracy and intra-batch precision of the developed method.

Caffeine concentrations in rat serum, plasma, and brains (ng/mL or ng/g) were determined using the LC-MS/MS method developed and validated in this study. The LC-MS/MS method consisted of a Shimadzu LC-20AD HPLC system (Kyoto, Japan), and chromatographic separation of the tested compound was achieved using a Waters XBridage reverse phase C18 column (5 cm × 2.1 mm I.D., packed with 3.5 μm) at 25 °C. Five microliters of the supernatant were injected. The flow rate of the gradient elution was 0.4 mL/min with mobile phase A (0.1% formic acid (Fischer Chemical (MI, USA)) in purified deionized water (Millipore purification system (MA, USA)) and mobile phase B (0.1% formic acid in acetonitrile). An AB Sciex QTrap 4500 mass spectrometer equipped with an electrospray ionization source (ABI-Sciex, Toronto, Canada) in positive-ion multiple reaction monitoring (MRM) mode was used for detection. Protonated molecular ions and the respective ion products were monitored at the transitions of m/z 195.2 > 138.1 for caffeine and 198.2 > 138.1 for the internal standard. We adjusted the instrument settings to maximize the analytical sensitivity and specificity of detection. Data were processed using Analyst software (version 1.6).

### Magnetic Resonance Image (MRI)

MRI scans were performed 24 h after HI 26. MRI was performed on an 11.7 Tesla (T) horizontal small-bore magnet (Biospec 117/16, Bruker, Billerica, MA, USA) using a rat brain receive only proton (1H) coil (Bruker Biospin). Anatomical images were acquired using a rapid acquisition relaxation enhancement (RARE) T2-weighted (T2W) sequence (echo time (TE) = 25 ms; repetition time (TR) = 2.9 s; inplane resolution 0.156 × 0.156 mm2). MRI images from anterior to posterior sagittal with a focus in Bregma 0.2 mm to -11.8 mm. The extent of the damage was scored according to the ordinal scale: 1= no damage, 2= mild damage, 3= moderate, and 4= several, based on the volume of the edema and area affected. MRI was used to decrease the number of animals for long-term behavior testing. A total of 27 animals were scanned (n=13 HI/Vehicle vs. n=14 HI/Caffeine). The median injury score was 4 for the HI/Vehicle group and 1.5 for the HI/Caffeine group. From the 27 animals scanned, we kept animals representing the median injury scores for long-term behavior testing; n=9 HI/Vehicle with a score of 3-4 and n=8 HI/Caffeine with a score of 1-2.

### Long-term behavior testing

The following behavioral tests were performed: Catwalk (P46-P49), and Novel Object Recognition (P53-P56). All tests were performed on three consecutive days, where the first two days were used as training.

The Catwalk is an assessment of motor function. We used the same setting regarding camera and recording as previously described 27. For a run to be considered successful, there must be a maximum run variation of <60% and completion within 5 s. Animals were habituated for two days with the Catwalk. On the third day, the animals were allowed to run through the catwalk and recorded three consecutive runs in the morning. Only those runs under 5 s were recorded. Animals were randomized between litters regarding the different treatments. XT gait analysis system (Noldus) was used to quantify the gait and locomotion parameters. All runs were first classified using automated footprint recognition in the Catwalk XT 10.6 software. Each run was manually reviewed and its annotation was corrected as needed by a blinded investigator. Each paw was labeled on the recorded video in order to calculate paw-related parameters. Gait parameters are represented as the median of three runs per animal per treatment. The gait-related parameters measured were the following: run speed (cm/s); step cycle (time (s) from when an initial paw gets in contact on the glass to the next time the paw comes in contact with glass); stand (duration (s) of contact of a paw with the glass plate), and swing speed (speed (distance unit/second) of the paw during swing). Each paw was compared individually between the treatment groups and no ratios (left/right) were calculated.

The Novel Object Recognition (NOR) test examined learning ability and memory, which was performed as previously described 28. Briefly, NOR was performed in four 45 × 45 cm boxes with white walls and a black floor. On the training days, each rat was placed in the same empty box for 5 min for habituation, with two identical objects placed in opposite corners. On day three, each rat was placed in the same box with two identical objects for 5 min. After a pause of 1h, each rat was placed in the same box where one of the objects was replaced by a novel object for another 5 min. The tests were recorded on video and analyzed using EthoVision XT 17.5 (Noldus Information Technology, Wageningen, Netherlands). The ratio of the total time each rat spent exploring either one of the two objects (c) to the time spent exploring the novel object (b) was calculated to determine the percentage of time the rat spent exploring the novel object (a) (a=(b/c) × 100).

### Immunohistochemistry

Following transcardiac perfusion with phosphate-buffered saline (PBS) and with 4% paraformaldehyde (Sigma-Aldrich), the brains were post-fixed in 4% paraformaldehyde overnight at 4 °C and embedded in paraffin. Immunohistochemistry was performed as previously described 29-31 using five animals per time point and group. We only used 24 h post HI brain samples for immunohistochemistry, as some of the areas in the ipsilateral brain were severely injured, and technical preparation of the brain regions was not feasible at later times (data not shown). After deparaffinization, 10 µm coronal sections (-3.8 ± 0.7 mm from Bregma) were rehydrated. Antigen retrieval was performed in pre-heated PBS 1x for 7 min following permeabilization with 0.1% Triton X-100 for 30 min at room temperature. After blocking with 20% normal goat serum in PBS 1x (Invitrogen, Germany), the slices were incubated with primary antibodies Supplementary Table 2 overnight at 4 °C, followed by incubation with the appropriate secondary antibody Alexa Fluor 488 or 594 for 1 h at room temperature. Both primary and secondary antibodies were diluted in 0.7% carrageenan solution with 0.02% NaN3 solution in PBS 1x. Sections were counterstained with 4,6-diamidino-2-phenylindole (DAPI) (Invitrogen). Immunohistochemistry was visualized by fluorescence microscopy (AxioScan Z. 1) using a 20x objective and confocal LSM900 (Zeiss, Germany) using a 20x objective with a zoom of 2-3x. The slices were analyzed using AxioScan.Z1. Three non-overlapping frames per each coronal section (approximately at -3.8 mm Bregma) were scanned per animal (Carl Zeiss Microscopy GmbH, Oberkochen, Germany). ZEN Blue 3.1 (Carl Zeiss Microscopy GmbH, Germany) and ImageJ software were used for analysis. The areas were standardized according to the degree of the damage in the vehicle group, particularly because the cortical area in our vehicle group is the most affected area (most damaged is the auditory cortical area), and the hippocampal areas often shrink due to the edema after HI as the ventricles expand and push upon the hippocampal region. Once these areas are defined in the vehicle animals, the same coordinates are taken in the treated samples. The areas analyzed were the cerebral cortex (particularly the primary somatosensory area including layer 2-5), and the hippocampus (particularly the CA2 and CA3 areas including the pyramidal layer, the stratum radiatum and stratum lacunosum-moleculares). A fixed area of 1000 x 1000 µm was drawn in the area of interest of the same coronal section (Bregma -3.8 mm). Z-stacking was performed using an interval of nine slices with a distance of 1 µm between each respective z-stack. Maximal projection was used to stich all z-stacks for posterior analyze and quantification of microglia, astrocytes, and neurons, where the cells positive for the corresponding antibody were matched with their respective nuclear staining, DAPI, and counted.

### Biochemistry analysis

The cortex and hippocampus were lysed in radioimmunoprecipitation (RIPA) buffer in addition to phosphatase and protease inhibitors. Total protein concentration was measured using a bicinchoninic acid assay (BCA, PierceTM Thermo Fisher) following the manufacturer's instructions. Immunoblotting was performed as previously described 31, with a few modifications. BIS-TRIS 10% gel was loaded with 50 µg total protein. SDS-polyacrylamide gel electrophoresis was performed in a 2-Morpholinoethanesulfonic acid buffer system (MES) buffer system (Thermo Fisher Scientific). The separated proteins were transferred onto a polyvinylidene difluoride membrane overnight at 4 °C and 30 V in a transfer buffer containing 10% methanol. The membranes were blocked in 5% (w/v) milk (for non-phosphorylated proteins) or 5% (w/v) bovine serum albumin (BSA) (for phosphorylated proteins) containing TBS with 0.05% (v/v) Tween 20 (MP Biomedical) for 1 h at room temperature, followed by incubation with the primary antibody Supplementary Table 2 overnight at 4 °C in 1% (v/v) blocking buffer. Secondary antibodies Supplementary Table 2, goat anti-mouse (IRDye 680 or IRDye 800), and goat anti-rabbit (IRDye 800), were used to develop the blots (LI-COR Biosciences) and imaged with an Odyssey infrared imaging system (LI-COR Biosciences). Optical density was determined using ImageJ software. A fix area was draw on the band of interest (right moleculare weight) and the area was measured. All areas were normalized to β-actin as a loading control. When it corresponded, the ratio between the total amount and the phosphorylated protein was determined. All protein bands were chosed based on the molecular weight (KDa) for the specific protein according to the data sheet for every antibody (red arrow head in the figures when it corresponds).

### Statistical analysis

**Figure 1:**
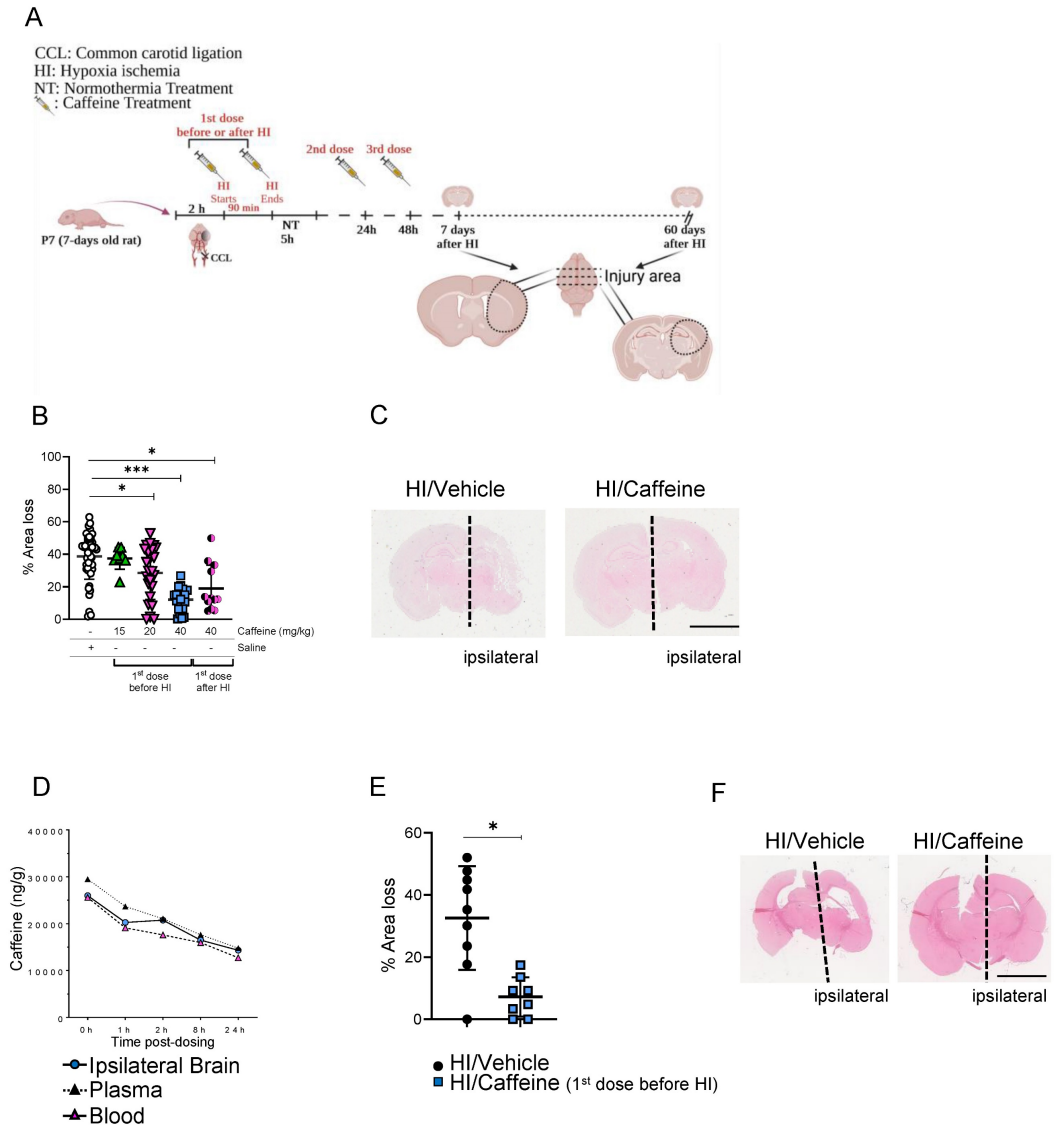
Neuroprotective effects of caffeine following HI treatment. **(A)** Schematic image of experimental design. P7 rat pups underwent ligation of the left common carotid artery followed by 90 min of hypoxia. Pups were randomized to different caffeine concentration following 5 h of NT (37 ◦C) after hypoxia. Two consecutive days of treatment with caffeine at the respective doses were administrated every 24 h. The animals were sacrificed after 7 days of survival. **(B)** Percentage of area loss showed a significant neuroprotective effect in animals treated with 40 mg/kg dose compared to the other dose and vehicle control, particularly when the first dose was before HI, with a reduction in the variability between animals compared to the concentration administered after HI. A dose of 120 mg/kg showed high mortality (data not shown). HI/Vehicle n=50 (white dot), HI/Caffeine (green triangles) (15 mg/kg) n=8, HI/Caffeine (pink triangles) (20 mg/kg) n=24, HI/Caffeine (40 mg/kg-first dose before HI - blue squares) n=23, and HI/Caffeine (40 mg/kg-first dose after HI - black/pink dots) n=11. One-way ANOVA followed by Tukey *post hoc* test for multiple comparison was used with *p<0.05 and ***p<0.0001. Data are expressed as the median (IQR). **(C)** Representative image of coronal section from HI/Vehicle vs. HI/Caffeine (40 mg/kg-before HI) staining with eosin showing the damage over the ipsilateral side on the HI/Vehicle treatment compared to HI/Caffeine treatment. **(D)** Pharmacokinetic study of blood, plasma, and ipsilateral brain samples after treatment with a single dose of caffeine 40 mg/kg before HI. A peak in the levels of caffeine in the samples was observed immediately after HI, showing a temporal decrease over 24 h. n=5 animals per time point. Ipsilateral brain (blue dot), plasma (black triangle) and blood (pink triangle). **(E)** Percentage of area loss 60-days after HI showed a significant neuroprotective effect in animals treated with 40 mg/kg dose compared to the vehicle. HI/Vehicle n=9 (black dot), HI/Caffeine n=8 (blue square). Nonparametric tests were performed using the Mann-Whitney U test with a 95% confidence interval with a *p<0.05. Data are expressed as the median (IQR). **(F)** Representative image of coronal section from HI/Vehicle vs. HI/Caffeine (40 mg/kg-before HI) staining with eosin showing the damage over the ipsilateral side on the HI/Vehicle treatment compared to HI/Caffeine treatment.

All numerical values are presented as median and interquartile range (IQR). All analyses and data plots were performed using GraphPad Prism 6 (GraphPad Software, La Jolla, CA, USA). For the analysis of protein activity (AMPK/mTOR/pathway) at different time points and comparing different treatments, (including inhibitors and caffeine), and for the analysis of area loss using different caffeine concentrations, the differences between groups were determined by one-way analysis of variance (one-way ANOVA) followed by Tukey post hoc test for multiple comparison with a 95% confidence interval. For Figure 1b, one-way analysis of variance (one-way ANOVA) followed by Tukey post hoc test for multiple comparison with a 95% confidence interval was used, while for Figure 1e, nonparametric tests were performed using the Mann-Whitney U test with a 95% confidence interval. For the behavioral tests, area loss, immunohistochemistry (Iba1+, GFAP+, NeuN+ cells), and protein expression at 4 and 24 h after HI (NeuN, CX3CR1, CCR2); nonparametric tests were performed using the Mann-Whitney U test with a 95% confidence interval, comparing the two treatment groups (HI/Vehicle vs. HI/caffeine). All animals were randomized between litters, sex and weight at the time of experiment. To determine the effects of sex and weight gain on the individual results, linear regression analysis was performed. In all of our results, statistical significance was set at ^*^p<0.05 or ^***^p<0.0001.

## Results

### Neuroprotective effect of caffeine after hypoxia-ischemia

To determinate the correct concentration of caffeine, we performed several experiments in which different concentrations were administered as a single intraperitoneal (i.p.) injection before or after HI and two consecutive doses at 24 and 48 h after the first dose (Fig. 1A). Analysis of area loss using hematoxylin and eosin staining showed that a 40 mg/kg dose significantly reduced area loss to 12.1 % when the first dose was administered before HI (Fig. 1B-C, ***p<0.0001. n=23; blue squares) or 19.03% when administered after HI (Fig. 1B-C, *p<0.05. n=11; black/pink dots), compared with 35.71 % area loss in the HI/Vehicle group (Fig. 1B-C. n=50; white dots). Notably, administration of 40 mg/kg before HI not only showed a significant reduction in the percentage of area loss compared to the HI/Vehicle group but also showed a reduction in the variability between individual animals (Fig. 1B), making this observation an important point. A significant neuroprotective effect was observed using 20 mg/kg, with a 28.5 % area loss compared to the vehicle-treated animals (Fig. 1B, *p<0.05. n=24; pink triangles). A lower dose of 15 mg/kg did not show a significant neuroprotective effect compared with vehicle-treated animals (Fig. 1B. n=8; green triangles). A much higher dose of 120 mg/kg was not well tolerated, and the animals did not survive the second dose of caffeine administered 24 h after HI Supplementary Table 1. Pharmacokinetic assays of samples at different time points after HI administration (40 mg/kg before HI) showed a high concentration of caffeine, with a mean peak in blood concentration of 29.5 mg/L (Fig. 1D, pink triangles), 25.6 mg/mL in the plasma (Fig. 1D, black triangles), and 25.98 mg/kg in the ipsilateral brain hemisphere (Fig. 1D, blue dots) immediately after HI (0 h after HI), which decreased over time to a concentration of 10.2 mg/mL in blood, 11.8 mg/mL in plasma and 11.4 mg/kg in the ipsilateral brain hemisphere 24 h after HI. These results demonstrate the high bioavailability of caffeine over the first 24 h after administration (Fig. 1D). Analysis of area loss 60-days after HI showed that a 40 mg/kg dose (first dose was administered before HI) significantly reduced area loss to 7.03 % compared with 35.3 % area loss in the HI/Vehicle group (Fig. 1E-F. *p<0.05, n=9 HI/Vehicle (black dots) and n=8 HI/Caffeine (blue squares)).

The effects of caffeine on sex were determinate by linear regression and non-parametric statistical analysis method 32. We did not observe sex differences between vehicle-treated animals and the different caffeine treatment groups, where the first dose of caffeine was administered before HI Supplementary Fig. 1A). We observed a significant reduction in the area loss in the female group compared to that in the male group when the first dose of caffeine was administered after HI (Supplementary Fig. 1A, *p<0.05). Analysis of sex correlation 60-days after treatment, where the first caffeine dose was administered before HI, did not show significant effects between both sexes (Supplementary Fig. 1B).

### Effect of caffeine on long-term functional outcome

**Figure 2:**
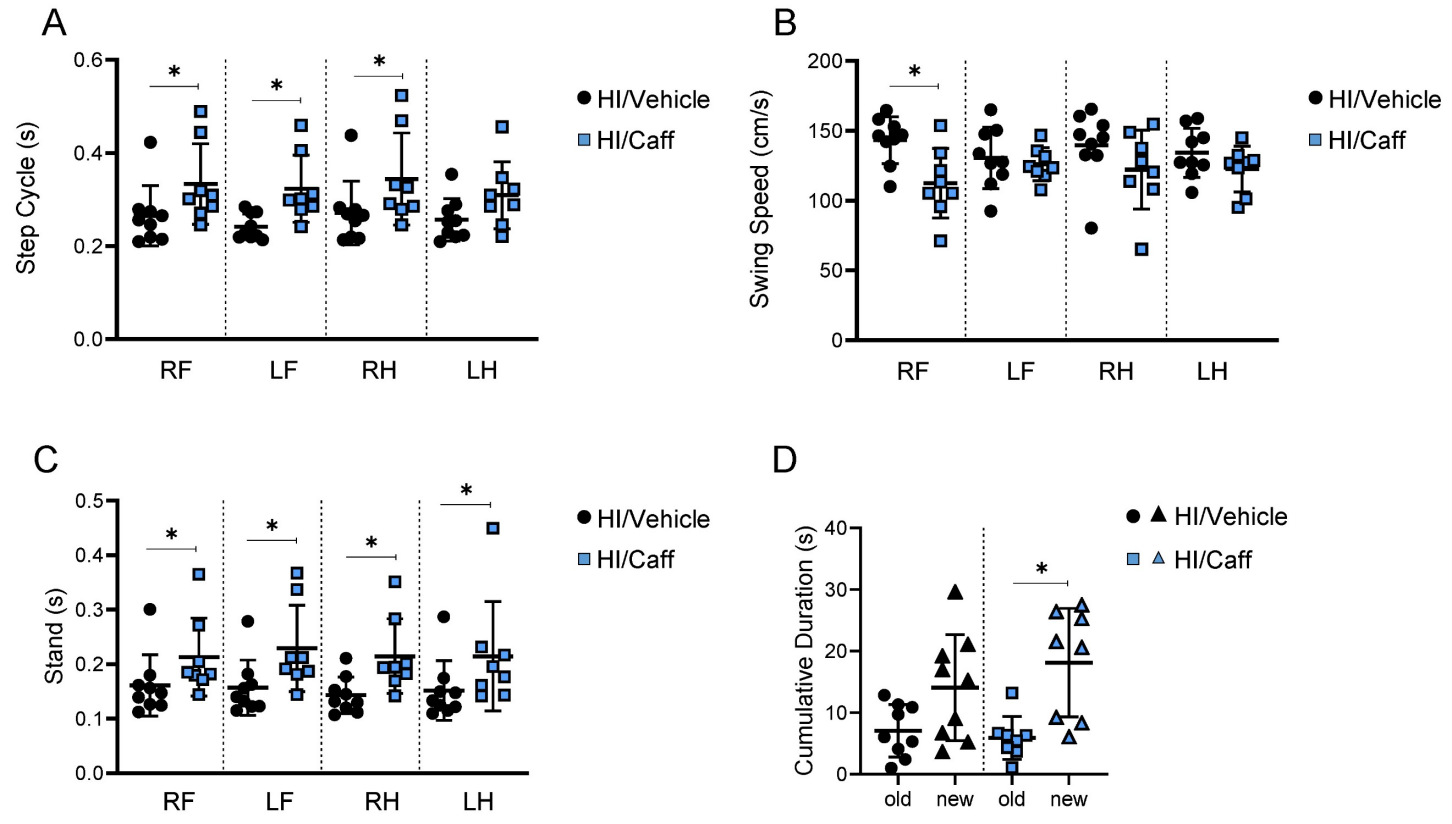
Long-term effects of caffeine on motor and cognitive behaviors. Motor behavior as Catwalk showed improvement in dynamic parameter of gait functions as **(A)** step cycle and **(B)** swing speed, as well as in static parameter of gait function as **(C)** stand. HI/Vehicle n=9 (black dots), HI/Caffeine n=8 (blue squares). **(D)** Cognitive behavior such as novel object recognition showed an increase in cumulative duration for the new object compared to old object after treatment with caffeine. HI/Vehicle n=9 (black dots-old object and black triangle-new object), HI/Caffeine n=8 (blue square-old object and blue triangle-new object). Nonparametric tests were performed using the Mann-Whitney U test with a 95% confidence interval with a *p<0.05. Data are expressed as median (IQR).

To assess the long-term outcome of caffeine treatment, we first preselected the animals using MRI 24 h after HI treatment with either vehicle or caffeine. The majority of the damage observed was from Bregma 0.2 mm to Bregma -11.8 mm (Supplementary Fig. 2A-C). The used injury score was based on the edema size and affected area, with 1 = no damage, 2 = mild damage, 3 = moderate damage, and 4 = severe damage. In animals scored 3-4, the edema was observed in the cortex (~ -9.8 mm from Bregma), striatal cortex (~ -8.3 mm), frontopariental motor and somatosensory cortex (~ -1.3 mm), and caudate putamen (~ 0.2 mm) (Supplementary Fig. 2A-C). A total of 27 animals were scanned (n=13 HI/Vehicle vs. n=14 HI/Caffeine). The median injury score was 4 for the HI/Vehicle group and 1.5 for the HI/Caffeine group. From the 27 animals scanned, we kept n=8 HI/Vehicle with a score of 3-4 (Supplementary Fig. 2B-C) and n=8 HI/Caffeine with a score of 1-2 (Supplementary Fig. 2B-C) for long-term behavior testing. The Catwalk assessment of motor function after HI in the rats treated with caffeine demonstrated a long-term improvement in behavioral parameters related to the front and hind paws, particularly for the dynamic parameters of gait function step cycles in both the right and left front limbs as well as the right hind limb in the HI/Caffeine group compared to the HI/Vehicle group (Fig. 2A, *p<0.05; HI/Vehicle black dots and HI/Caffeine: blue squares). Another dynamic parameter, swing speed, showed improvement only in the right front limb in the HI/Caffeine group compared to that in the HI/Vehicle group (Fig. 2B, *p<0.05; HI/Vehicle black dots and HI/Caffeine: blue squares). The static parameters of gait function as stand showed a significant improvement in the four limbs in the HI/Caffeine group compared to the HI/Vehicle group (Fig. 2C, *p<0.05; HI/Vehicle black dots and HI/Caffeine: blue squares). In the assessment of cognitive function after HI using NOR, we observed that the animals treated with caffeine after HI showed a significant interest in the novel object compared with the vehicle animals (Fig. 2D, *p<0.05; HI/Vehicle black dots and HI/Caffeine: blue squares).

We did not observe a significant difference between the sexes in the effects of caffeine on long-term functional outcomes (data not shown).

### Neuroprotective effects of caffeine in the brain after hypoxia-ischemia

**Figure 3:**
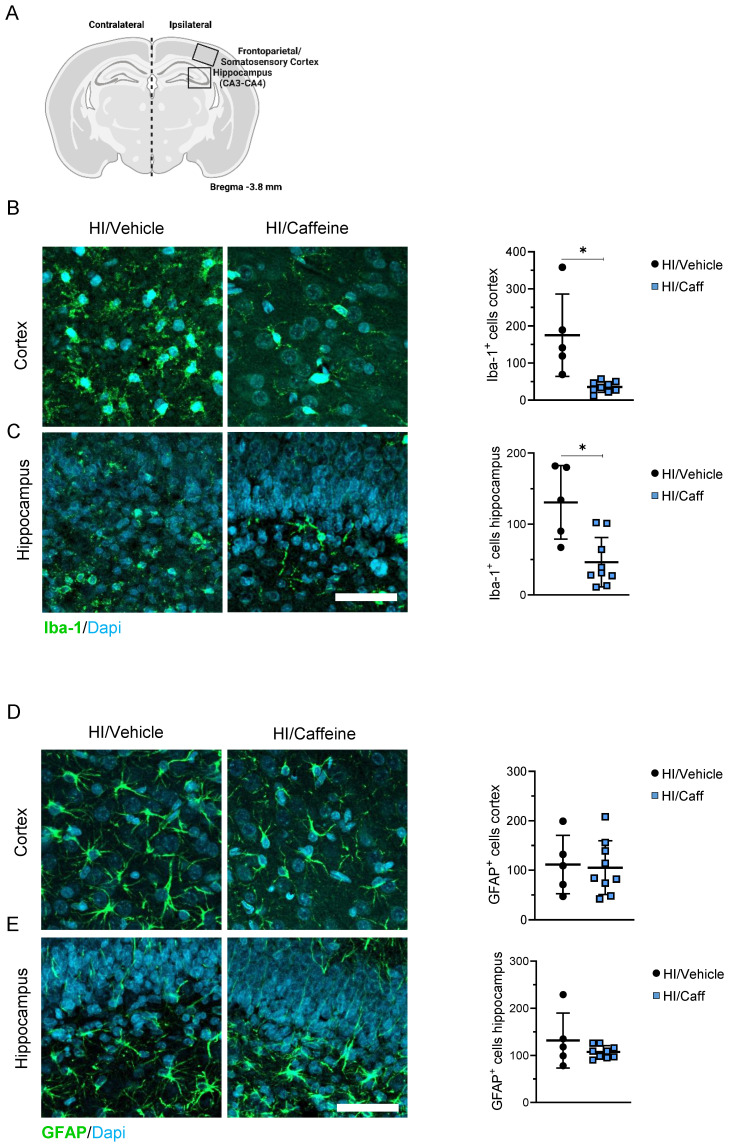
Effect of caffeine on gliosis following HI treatment. **(A)** Schematic image of areas in the cortex and hippocampus used for quantitative analysis. **(B)** Representative image and quantification of the cortical area staining for the microglia marker Iba-1 for HI/Vehicle and HI/Caffeine groups. Green Iba-1 and nuclear marker DAPI in blue. **(C)** Representative image and quantification of the hippocampal area staining for the microglia marker Iba-1 for HI/Vehicle and HI/Caffeine groups. Green Iba-1 and nuclear marker DAPI in blue. **(D)** Representative image and quantification of the cortical area staining for the astrocyte marker GFAP Iba-1 for HI/Vehicle and HI/Caffeine groups. Green GFAP and nuclear marker DAPI in blue. **(E)** Representative image and quantification of the hippocampal area staining for the astrocyte marker GFAP Iba-1 for HI/Vehicle and HI/Caffeine groups. Green GFAP and nuclear marker DAPI in blue. HI//Vehicle n=5 (black dots), HI/Caffeine n=9 (blue squares). Nonparametric tests were performed using the Mann-Whitney U test with a 95% confidence interval with a *p<0.05. Data are expressed as median (IQR). Scale bar 20 µm. Image **A** made with Biorender.com.

We characterized the neuroprotective effects of caffeine on microgliosis and astrogliosis in the most affected areas after HI, the cortex, and the hippocampus (Fig. 3A). First we determined the effect of caffeine on microgliosis. We observed that animals treated with caffeine 40 mg/kg before HI, following a single dose at 24 and 48 h, showed a significant reduction in microgliosis (reduction in Iba-1+ cells) in the cortex and hippocampus 7-days after HI compared to the HI/Vehicle group (Fig. 3B-C, *p<0.05; HI/Vehicle: black dots and HI/Caffeine: blue squares). To determine early microglia and monocytes-derived macrophages activation at early time points, we determined the protein levels of Iba-1, CX3CR1 (a microglia surface marker), and monocytes-derived macrophage CCR2 using western blotting at 4 and 24 h after HI in the cortex and hippocampus (Supplementary Fig. 3A). In the cortical area, a significant increase in Iba-1 was observed in the HI/Caffeine group compared to the HI/Vehicle group 4 h after HI (Supplementary Fig. 3B, *p<0.05; HI/Vehicle: black dots and HI/Caffeine: blue squares); however, twenty-four hours after HI, Iba-1 in the HI/Caffeine group was significantly reduced compared to the HI/Vehicle group (Supplementary Fig. 3B, *p<0.05; HI/Vehicle: black dots and HI/Caffeine: blue squares). Interestingly, CX3CR1 were significantly reduced in the HI/Caffeine group compared to the HI/Vehicle group 4 h after HI (Supplementary Fig. 3C, *p<0.05; HI/Vehicle: black dots and HI/Caffeine: blue squares). Analysis of CCR2 did not show significant differences in the cortex at either time point analyzed (Supplementary Fig. 3D, *p<0.05; HI/Vehicle: black dots and HI/Caffeine: blue squares). The hippocampus showed different pattern. For Iba-1, no significant changes were observed at either time point (Supplementary Fig. 3E, *p<0.05; HI/Vehicle: black dots and HI/Caffeine: blue squares). CX3CR1 showed a significant reduction in the HI/Caffeine group compared to the HI/Vehicle group at both time points analyzed (Supplementary Fig. 3F, *p<0.05; HI/Vehicle: black dots and HI/Caffeine: blue squares). Interestingly, the same effect was observed with CCR2, with a significant reduction in the HI/Caffeine group compared to the HI/Vehicle group at both time points (Supplementary Fig. 3G, *p<0.05; HI/Vehicle: black dots and HI/Caffeine: blue squares).

To determine the effect of caffeine on astrogliosis, GFAP positive-cells were measured in the cortex and hippocampus 7-days after HI. We did not observe changes in astrogliosis in the HI/Caffeine groups compared to the HI/Vehicle group 7-days after HI in either area analyzed (Fig. 3D-E, *p<0.05; HI/Vehicle: black dots and HI/Caffeine: blue squares). To determine the early expression levels of GFAP at early time points, we performed western blotting at 4 and 24 h after HI in the cortex and hippocampus (Supplementary Fig. 4A). In both areas analyzed we observed a significant reduction in GFAP in the HI/Caffeine group compared to the HI/Vehicle group 24 h after HI (Supplementary Fig. 4B and C, *p<0.05; HI/Vehicle: black dots and HI/Caffeine: blue squares).

**Figure 4:**
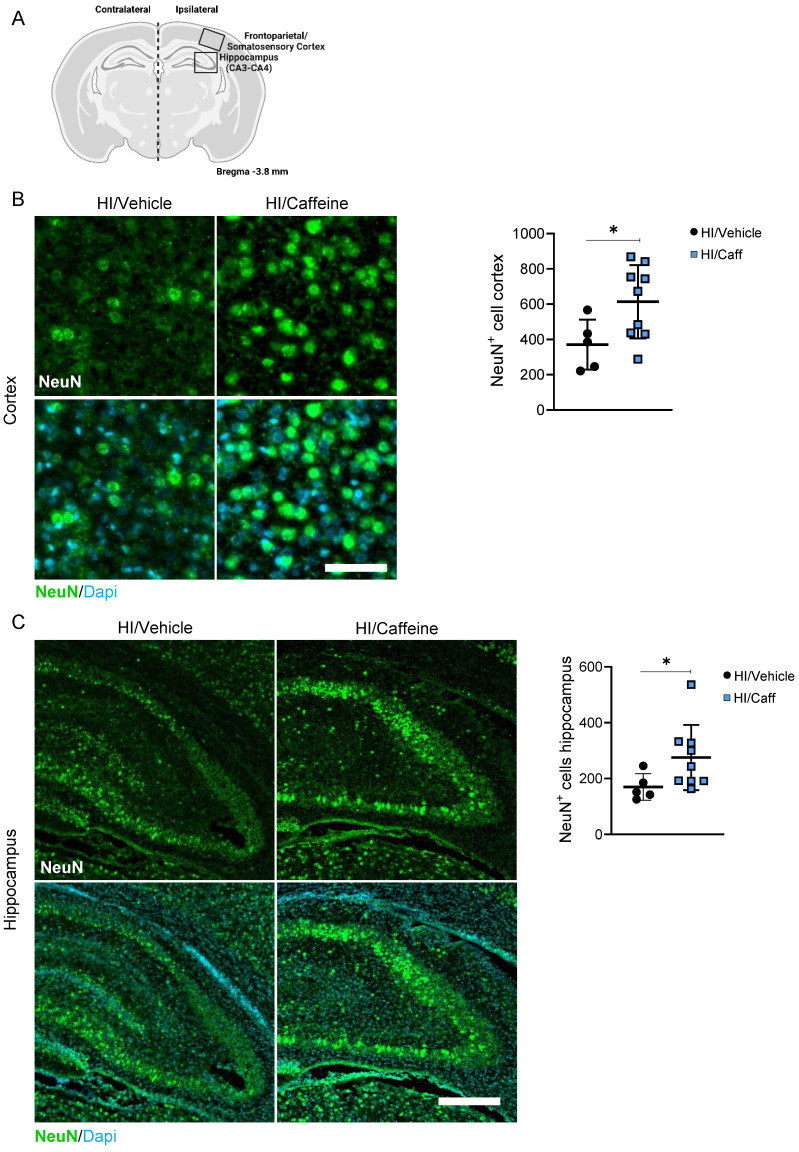
Effect of caffeine on the number of neurons following HI treatment. **(A)** Schematic image of areas in the cortex and hippocampus used for quantitative analysis. **(B)** Representative image and quantification of the cortical area staining for the neuronal marker NeuN for HI/Vehicle and HI/Caffeine groups. Green NeuN and nuclear marker DAPI in blue. **(C)** Representative image and quantification of the hippocampal area staining for the neuronal marker NeuN for HI/Vehicle and HI/Caffeine groups. Green NeuN and nuclear marker DAPI in blue. HI/Vehicle n=5 (black dots), HI/Caffeine n=9 (blue squares). Nonparametric tests were performed using the Mann-Whitney U test with a 95% confidence interval with a *p<0.05. Data are expressed as median (IQR). Scale bar 20 µm (B), 100 µm (D). Image **A** made with Biorender.com.

Analysis of the effect of caffeine on the neuronal layer in the cortical and hippocampal areas after caffeine treatment (Fig. 4A) showed a significant increase in the number of positive NeuN neurons in the somatosensory cortex and the CA3-CA4 areas of the hippocampus 7-days after HI in the HI/Caffeine group compared to that in the HI/Vehicle group (Fig. 4B-C, *p<0.05; HI/Vehicle: black dots and HI/Caffeine: blue squares). To determine early expression levels of NeuN at early time points, we performed western blotting at 4 and 24 h after HI in the cortex and hippocampus (Supplementary Fig. 5A). In the cortical area, a significant reduction in NeuN was observed in the HI/Caffeine group compared to the HI/Vehicle group 24 h after HI (Supplementary Fig. 5B, *p<0.05; HI/Vehicle: black dots and HI/Caffeine: blue squares). No significant changes were observed in the hippocampus at either time point (Supplementary Fig. 5C; HI/Vehicle: black dots and HI/Caffeine: blue squares).

### AMPK/mTOR protein activity following hypoxia ischemia

**Figure 5:**
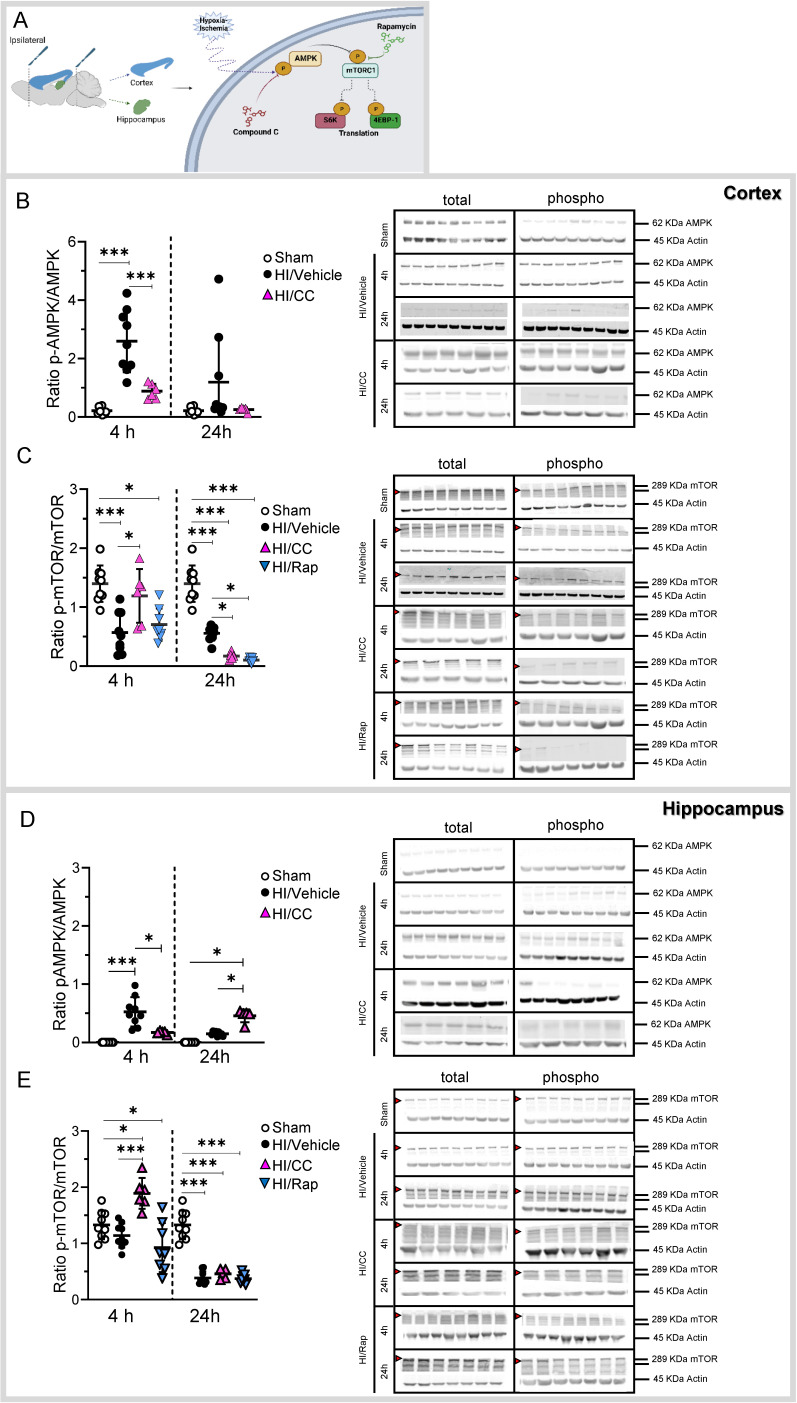
AMPK/mTOR protein activity following HI treatment. **(A)** Schematic image showing the dissection of the ipsilateral cortex and hippocampus (left): Schematic picture showing the molecular pathway hypothesized in the activation/inhibition of the AMPK/mTOR pathway following HI or after HI treatment with either the specific inhibitor of AMPK (Compound C-CC) or mTOR (Rapamycin-Rap) (right). **(B)** Protein level is shown as the ratio of phosphorylated AMPK to total AMPK in the cortex. Representative protein bands of a western blot for AMPK for the sham, HI/Vehicle, HI/CC, and HI/Rap at 4 and 24 h at the cortex. Actin indicates equal protein loading. **(C)** Protein level is shown as the ratio of phosphorylated mTOR to total mTOR in the cortex. Representative protein bands of a western blot for mTOR for the sham, HI/Vehicle, HI/CC, and HI/Rap at 4 and 24 h at the cortex. Actin indicates equal protein loading. Arrow head point mTOR band analyzed. **(D)** Protein level is shown as the ratio of phosphorylated AMPK to total AMPK in the hippocampus. Representative protein bands of a western blot for AMPK for the sham, HI/Vehicle, HI/CC, and HI/Rap at 4 and 24 h at the hippocampus. Actin indicates equal protein loading. **(E)** Protein level is shown as the ratio of phosphorylated mTOR to total mTOR in the hippocampus. Representative protein bands of a western blot for mTOR for the sham, HI/Vehicle, HI/CC, and HI/Rap at 4 and 24 h at the hippocampus. Actin indicates equal protein loading. Arrow head point mTOR band analyzed. Sham n=9 (white dot), HI/Vehicle n=9 (black dot), and HI/Rap (blue triangles) n=8 at both time points. HI/CC (pink triangles) n=6 at 4 h, n=5 at 24 h. One-way ANOVA followed by Tukey *post hoc* test for multiple comparison was used with *p<0.05 and ***p<0.0001. Data are expressed as median (IQR). Image **A** made with Biorender.com.

To demonstrate the role of the AMPK/mTOR pathway in our neonatal hypoxia-ischemia model, we studied the activity (ratio between the phosphorylated (active form) and non-phosphorylated (non-active form) of the protein) at different time points (4 and 24 h after HI) in the cortical and hippocampal areas (Fig. 5A and Supplementary Table 3 using western blotting.

In the cortex, we found a significant increase in the AMPK activity 4 h after HI compared to the sham group (Fig. 5B, ***p<0.0001; sham white dots, HI/Vehicle black dots). We used a specific inhibitor of the AMPK pathway to demonstrate its role in HI (Compound C-Supplementary Table 1)). Compound C showed a significant inhibitory effect on AMPK in the cortex 4 h after HI (Fig. 5B, ***p<0.0001; HI/CC pink triangles). In contrast, mTOR activity in the cortical area was significantly decreased at 4 and 24 h after HI compared to that in the sham group (Fig. 5C, ***p<0.0001; sham white dots, HI/Vehicle black dots). In addition, we used a specific inhibitor of the mTOR pathway, rapamycin, to demonstrate its role in HI Supplementary Table 1. Rapamycin treatment showed a significant inhibitory effect on mTOR in the cortical area 4 h after treatment compared to the sham group (Fig. 5C, *p<0.05; HI/Rap blue triangles) and at 24 h after treatment compared to the sham and HI/Vehicle groups (Fig. 5C, ***p<0.0001 and *p<0.05, respectively; HI/Rap blue triangles).

In the hippocampus, we found a significant increase in AMPK 4 h after HI compared to the sham group (Fig. 5D, ***p<0.0001; sham white dots, HI/Vehicle black dots). Compound C showed a significant inhibitory effect on AMPK in the hippocampal area 4 h after HI compared to the HI/Vehicle group (Fig. 5D, *p<0.05; HI/CC pink triangle). Twenty-four hours after HI, a significant increase in AMPK was observed in the hippocampal area after treatment with compound C compared to the sham and HI groups (Fig. 5D, *p<0.05; HI/CC pink triangle). In contrast, mTOR activity was significantly decreased in the hippocampal area 24h after HI compared to that in the sham group (Fig. 5E, ***p<0.0001; HI/Vehicle black dots). Compound C showed a significant increase in mTOR in the hippocampal area 4 h after HI compared to the sham and HI/Vehicle groups (Fig. 5E, *p<0.05, and ***p<0.0001, respectively; HI/CC pink triangle). However, compound C showed a significant inhibitory effect 24 h after HI compared with the sham group (Fig. 5E, ***p<0.0001; HI/CC pink triangle). Rapamycin treatment significantly inhibited mTOR activity in the hippocampal area at both time points compared to the sham group (Fig. 5E, *p<0.05 (4 h) and ***p<0.0001 (24 h); HI/Rap blue triangles).

Sex correlation analysis of the AMPK/mTOR pathway did not show significant differences between the sexes Supplementary Fig. 1C-F).

### Down-stream mTOR-regulated protein activity following hypoxia ischemia

mTOR activity regulates two main downstream regulators which play a key role in translation: S6 and 4EBP-1 (33. We measured the activity (ratio between the phosphorylated (active form) to non-phosphorylated (non-active form) for of the protein) of S6 and 4EBP-1 using western blotting at both time points in the cortical and hippocampal areas Supplementary Table 3.

In the cortical area, we observed a significant decrease in 4EBP-1 in the HI/Vehicle group compared to that in the sham group 24 h after HI Supplementary Fig. 6A, ***p<0.0001; sham: white dots and HI/Vehicle: black dots). The use of compound C resulted in a significant increase in 4EBP-1 compared to both the sham and HI/Vehicle groups 4 h after HI; however, a significant decrease was observed in 4EBP-1 24 h after HI compared to that in the sham group (Supplementary Fig. 6A, ***p<0.0001; HI/CC pink triangle). We observed a significant decrease in 4EBP-1 24 h after rapamycin treatment compared to the sham group (Supplementary Fig. 6A, ***p<0.0001, HI/Rap blue triangles). Interestingly, S6 was significantly downregulated in the HI/Vehicle group 4 h after HI compared to those in the sham group (Supplementary Fig. 6B, ***p<0.0001; HI/Vehicle black dots). Treatment with compound C resulted in S6 activity similar to those in the sham group (not significant) 4 h after HI, but it was significantly increased compared to that in the HI/Vehicle group (Supplementary Fig. 6B, ***p<0.0001; HI/CC pink triangles). However, 24 h after treatment, the levels of S6 remained significantly lower than those in the sham and HI/Vehicle groups (Supplementary Fig. 6B, ***p<0.001; HI/CC pink triangles). Rapamycin treatment significantly increased the activity of S6 compared to HI at 4 h but remained below the sham values after treatment (Supplementary Fig. 6B, *p<0.05; HI/Rap blue triangles). Twenty-four hours after treatment, S6 remained significantly lower than those in the sham or HI/Vehicle groups (Supplementary Fig. 6B, ***p<0.0001; HI/Rap blue triangles).

**Figure 6:**
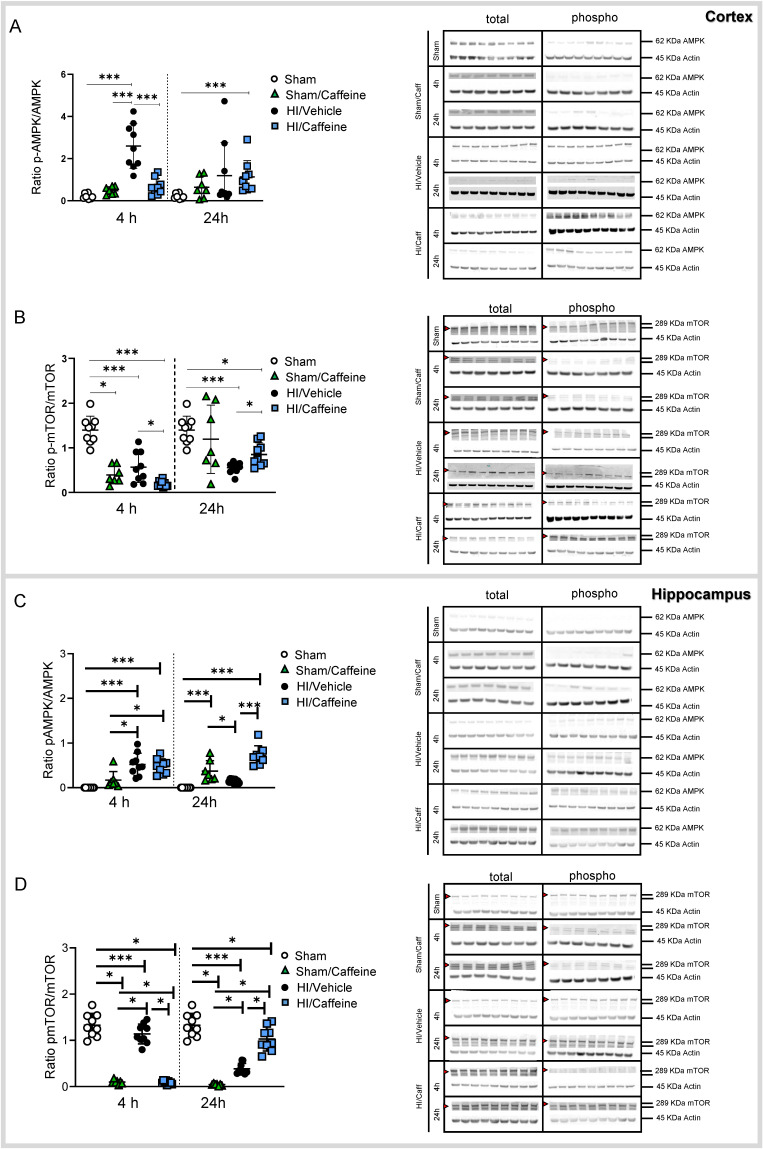
Caffeine effect on the AMPK/mTOR pathway protein activity following HI treatment. **(A)** Protein level was expressed as the ratio of phosphorylated AMPK to total AMPK in the cortex. Representative protein bands of a western blot for AMPK for the Sham, Sham/Caffeine, HI/Vehicle and HI/Caffeine groups in the cortex. Actin indicates equal protein loading. **(B)** Protein level was expressed as the ratio of phosphorylated mTOR to total mTOR in the cortex. Representative protein bands of a western blot for mTOR for the HI/Vehicle and HI/Caffeine groups in the cortex. Actin indicates equal protein loading. Arrow head point mTOR band analyzed.** (C)** Protein level was expressed as the ratio of phosphorylated AMPK to total AMPK in the hippocampus. Representative protein bands of a western blot for AMPK for the HI/Vehicle and HI/Caffeine groups in the hippocampus. Actin indicates equal protein loading** (D)** Protein level was expressed as the ratio of phosphorylated mTOR to total mTOR in the hippocampus. Representative protein bands of a western blot for mTOR for the HI/Vehicle and HI/Caffeine groups in the hippocampus. Actin indicates equal protein loading. Arrow head point mTOR band analyzed. Sham n=9 (white dots), Sham/Caffeine n=7 (green triangles), HI/Vehicle n=9 (black dots), and HI/Caffeine n=9 (blue squares) for each time points. One-way ANOVA followed by Tukey *post hoc* test for multiple comparison was used with *p<0.05 and ***p<0.0001. Data are expressed as median (IQR).

The hippocampus showed a different pattern. 4EBP-1 showed a significant decrease compared to the sham group at both time points (Supplementary Fig. 6C, *p<0.05 (4 h) and ***p<0.0001 (24 h); HI/Vehicle black dots). Compound C resulted in a significant increase in 4EBP-1 compared to the sham and HI/Vehicle groups 4 h after HI (Supplementary Fig. 6C, *p<0.05, and ***p<0.0001, respectively; HI/CC pink triangles). However, compound C-treated group showed a significant decrease 24 h after HI compared to the sham group (Supplementary Fig. 6C, *p<0.05, HI/CC pink triangles). The Rapamycin treated group showed a significant increase 24 h after HI compared to that in the HI/Vehicle group (Supplementary Fig. 6C, ***p<0.001; HI/Rap blue triangles). No significant changes were observed for S6 after compound C treatment compared to the sham group at either time point (Supplementary Fig. 6D; HI/Vehicle black dots). Compound C showed a significant increase compared to the sham group 4 h after HI (Supplementary Fig. 6D, *p<0.05; HI/CC pink triangles) or compared to the sham and HI/Vehicle groups 24 h after HI (Fig. 6D, ***p<0.0001; HI/CC pink triangles). Rapamycin treatment significantly decreased 24 h after HI compared to that in the sham and HI/Vehicle groups (Supplementary Fig. 6D, *p<0.05; HI/Rap blue triangles).

### Caffeine Treatment regulates the AMPK/mTOR pathway after hypoxia-ischemia

To elucidate the effect of caffeine on the AMPK/mTOR pathway, we performed western blotting of brain samples from the cortex and hippocampus on the ipsilateral side of the brain after HI (Supplementary Table 3.

In the cortex, AMPK was significantly increased in the HI/Vehicle group compared to that in the Sham and Sham/Caffeine groups 4h after HI (Fig. 6A, ***p<0.0001; Sham white dots, Sham/Caffeine green triangles, HI/Vehicle black dots). However, after caffeine treatment, AMPK was significantly reduced in the HI/Caffeine group compared to the HI/Vehicle group 4 h after HI (Fig. 6A, ***p<0.0001; HI/Vehicle: black dots and HI/Caffeine: blue squares). However, a significant increase in AMPK was observed in the HI/Caffeine group compared to the Sham group 24 h after HI (Fig. 6A, ***p<0.0001; Sham white dots, HI/Caffeine blue squares). mTOR showed a significant decrease in all groups compared to the Sham group, particularly in the HI/Caffeine group compared to the HI/Vehicle group 4 h after HI (Fig. 6B, *p<0.05 and ***p<0.0001, respectively; Sham white dots, Sham/Caffeine green triangles, HI/Vehicle black dots, and HI/Caffeine blue squares). Twenty-four hours after HI, a significant decrease was observed in the HI/Vehicle and HI/Caffeine groups compared to the Sham group; however, the HI/Caffeine group showed a significant increase compared to the HI/Vehicle group (Fig. 6B, *p<0.05 and ***p<0.0001, respectively; HI/Vehicle black dots and HI/Caffeine blue squares).

In the hippocampus, AMPK was significantly increased in the HI/Vehicle and HI/Caffeine groups compared to that in the Sham and Sham/Caffeine groups 4 h after HI (Fig. 6C, ***p<0.0001 and *p<0.05, respectively; Sham white dots, Sham/Caffeine green triangles, HI/Vehicle black dots, and HI/caffeine blue squares). However, 24 h after HI, the Sham/Caffeine and HI/Caffeine groups showed a significant increase in AMPK compared to the Sham group, while only HI/Caffeine showed a significant increase compared to the HI/Vehicle group (Fig. 6C, ***p<0.0001 and *p<0.05, respectively; Sham white dots, Sham/Caffeine green triangles, HI/Vehicle black dots, and HI/Caffeine blue squares). mTOR levels were significantly decreased in the Sham/Caffeine and HI/Caffeine groups compared to the Sham and HI/Vehicle groups 4 h and 24 h after HI, whereas 24 h after HI, a significant increase was observed in the HI/Caffeine group compared to that in the HI/Vehicle group (Fig. 6D, ***p<0.0001 and *p<0.05, respectively; Sham white dots, Sham/Caffeine green triangles, HI/Vehicle black dots, and HI/caffeine blue squares).

Translation factors 4EBP1 and S6 showed different expression patterns. In the cortex, 4EBP-1 was significantly reduced in the Sham/Caffeine and HI/Caffeine group compared with that in the Sham and HI/Vehicle group 4 h after HI, while 24 h after HI all groups were significate reduced compared to the Sham group Supplementary Fig. 7A, ***p<0.0001 and *p<0.05 respectively; Sham white dots, Sham/Caffeine green triangles, HI/Vehicle black dots and HI/Caffeine blue squares). S6 shows a significant decrease in all groups compared to the Sham group 4 h after HI; however, HI/caffeine showed a significant increase compared to the HI/Vehicle group (Supplementary Fig. 7B, ***p<0.0001 and *p<0.05, respectively; Sham white dots, Sham/Caffeine green triangles, HI/Vehicle black dots, and HI/caffeine blue squares). Twenty-four hours after HI, Sham/Caffeine and HI/Caffeine groups showed a significate decrease compared to the Sham and HI/Vehicle groups (Supplementary Fig. 7B, ***p<0.0001 and *p<0.05 respectively; Sham white dots, Sham/Caffeine green triangles, HI/Vehicle black dots and HI/Caffeine blue squares).

In the hippocampus, 4EBP-1 was significantly reduced 4 h after HI in all groups compared to the Sham group and in the HI/caffeine group compared to the HI/Vehicle group, while 24 h after HI, all groups were significantly decreased compared to the Sham group (Supplementary Fig. 7C, ***p<0.0001 and *p<0.05, respectively; Sham white dots, Sham/Caffeine green triangles, HI/Vehicle black dots, and HI/caffeine blue squares). S6 only showed significant increases in the HI/Vehicle and HI/Caffeine groups compared to the Sham and Sham/Caffeine groups, particularly in the HI/Caffeine group compared to the HI/Vehicle group 4 h after HI (Supplementary Fig. 7D, ***p<0.0001 and *p<0.05, respectively; Sham white dots, Sham/Caffeine green triangles, HI/Vehicle black dots, and HI/caffeine blue squares).

Sex correlation analysis of the AMPK/mTOR pathway did not show significant differences between sexes (Supplementary Fig. 1C-F).

## Discussion

In the present study, we demonstrated the neuroprotective effects of caffeine at a dose of 40 mg/kg in a neonatal model of hypoxic-ischemic brain injury. The neuroprotective effect of caffeine has been shown to reduce inflammation and increase the number of neurons in the cortical and hippocampal areas of the injured brain. The present study revealed that neonatal hypoxia-ischemia is associated with the activation of the AMPK/mTOR signaling pathway, which we demonstrated to be time- and brain-area-dependent. We proved that inhibition of the AMPK/mTOR pathway by compound C or rapamycin significantly affected protein regulation in our model, and similar effects were observed after caffeine treatment, demonstrating that the neuroprotective effects of caffeine regulate the AMPK/mTOR pathway.

At the cellular level, HIE is associated with a decrease in oxygen levels, leading to ATP depletion and compromised autophagy (34-36. When mTOR is activated, it stimulates a number of proteins and enzymes involved in anabolic processes under physiological glucose concentrations, while restricting the autophagy process. Conversely, when glucose levels are low, mTOR is inhibited by AMPK, leading to the repression of numerous anabolic processes, sparing ATP and antioxidants and increasing autophagy activity 37-30.

The majority of studies on AMPK/mTOR mechanisms following hypoxic-ischemic injury have utilized adult models of stroke, variations in the neonatal Vannucci model of HI, or *in vitro* models of HI 40-45. However, these results cannot be easily translated into the neonatal brain environment, and many molecular mechanisms differ between the immature and adult brains. Along these lines, a deeper understanding of the molecular mechanisms triggered by HI will provide better opportunities to discover new therapeutic targets for neonatal HIE.

In the present study, we observed the most pronounced changes in the activity pattern of AMPK/mTOR 4 h after HI in both areas analyzed, whereas 24 h after HI, the changes observed showed clear variation between the cortex and hippocampus. We observed a clear activation of AMPK and a reduction in mTOR levels immediately after HI. Partial inactivation of mTOR was observed at the level of both downstream S6 and 4EBP-1 in both areas analyzed. AMPK is highly expressed in the cortical and hippocampal neurons under ischemic stress 24, 41. However, the exact role of AMPK after an insult remains controversial. It has been demonstrated that the activation of AMPK protects hippocampal neurons, and reduce gliosis after glucose deprivation 43, 46, 47. Williams *et al.*, has demonstrated that AMPK activity is not required for neural development, but AMPK over activation during metabolic stress impairs neuronal polarization due to mTOR-inhibition 48. On contrast, the use of enhancers for AMPK protects against global cerebral ischemia with a significant reduction in neuronal death, apoptosis, and an increase in mitochondrial biogenesis 49, 50. mTOR participates in the regulation of HIF-1α, VEGF, and neuronal apoptosis, serving neuroprotective functions after HI in the developing rat brain, while inhibition of mTOR by rapamycin increased neuronal apoptosis 51. However, it has been shown that inhibition of mTOR by rapamycin in an adult model of stroke suppresses autophagy, prevents cytochrome c release, reduces ischemic brain damage 52, and improves motor impairments 53. At the moment, there are controversial results regarding the use of compound C or rapamycin as neuroprotective agents regulating the AMPK/mTOR pathway after energy stress. However, most of the available data indicate that inhibition of AMPK/mTOR is time- and concentration-dependent and relays when the first dose is administered. Another important point, is how the different areas in the brain response to the insult, demonstrating the complexity of the molecular pathways and potential compensation by other molecular mechanisms 54, 55 without discarding regional differences due to the diversity in vascularity distribution in the brain and differences in metabolic activity 56. The challenge remains to achieve tissue- and context-specific alterations, which depend on the temporal evolution of the insult in AMPK/mTOR activity, given the far-reaching and perhaps undesirable consequences of global AMPK/mTOR modulation.

In this study, we demonstrate the potential of caffeine as a neuroprotective agent. A single daily dose (before HI, and 24 h and 48 h after HI) showed significant neuroprotective effects, reduced brain area loss, decreased microgliosis, and increased the number of neurons in the brain. We also demonstrated that caffeine played a key role in the AMPK/mTOR pathway. Caffeine significantly reduced AMPK/mTOR activity in the cortex and hippocampus in a time-dependent manner. Because the analysis was performed before the administration of the second dose of caffeine, this effect is most likely a response to the pharmacokinetics of caffeine in rats. Our data revealed clear areas and time-dependent effects of caffeine on the AMPK/mTOR pathway. In the long term, we found that caffeine reduced the extent of the insult by reducing area loss, not only immediately after the insult but also over a period of a month after HI, which has a clear effect on the improvement of motor and cognitive functions. Caffeine reduced microgliosis in our animals with clear time- and area -dependent effects not only in microglia cells but also in monocytes-derived macrophages after caffeine treatment. Interestingly, we observed a reduction in astrogliosis twenty-four hours after caffeine treatment, but not in the long term (7-days after HI). These results showed that caffeine plays a key role in gliosis immediately after HI; however, caffeine showed to have a major role on microglial inflammatory effects following HI. Microglial cells (resident cells from the brain) and monocytes-derived macrophage (infiltrated immune cells from the periphery) play an important role in inflammatory resolution after HI, due to their pro- and anti-inflammatory responses 57. Microglial depletion in a neonatal model of HI aggravated neuronal damage and apoptosis after HI insult 58. In an adult model of stroke, resident microglia adopt a pro-inflammatory phenotype after stroke, and the infiltration of monocytes is involved in the early debris clearance of dying cells 59. The resolution of inflammation requires in-depth understanding how tissue-resident leukocytes respond differently to an injury depending on their site of residence and how this can be pruned into an anti-inflammatory effect 60, 61. We also observed a clear increase in the number of neurons following caffeine treatment. Long-term analysis of neurogenesis will present a limitation based on the extent of damage presented by the vehicle group compared with caffeine-treated animals. This limitation makes it difficult to compare neuronal proliferation between the two treatments in a reliable and comparative study based on the full extent of damage. However, further experiments are required to understand the effects of caffeine on neurogenesis.

For decades, caffeine has been used to treat apnea of prematurity. Caffeine is hydrophilic, diffuses freely into intracellular tissue water in all biological fluids with a high volume of distribution, is sufficiently lipophilic to pass through all biological membranes, readily crosses the blood-brain barrier with similar concentrations in serum and cerebrospinal fluid, can cross the placenta by passive diffusion, and is widely distributed in fetal tissues 62. Notably, caffeine is cost-effective and has a reduced need for drug monitoring. Caffeine is the preferred treatment, in part because of its long half-life and broad therapeutic profile, including in the brain, by reducing inflammation and improving myelination 15, 63. It has been safely approved after the “Caffeine for Apnea of Prematurity (CAP) trial” 64, 65, demonstrating that it not only reduced the incidence of bronchopulmonary dysplasia (BPD), but also in an 18-months follow-up study that revealed a neuroprotective outcome associated with a lower incidence of cerebral palsy and cognitive delay, and reduced motor disability without affecting intelligence, attention, or behavior 16. Four decades of use in high-income countries has confirmed the low-risk profile of caffeine in neonates and its ease of administration. It is one of the most widely prescribed drugs in neonatal units in high-income settings and could be a potential treatment for neonatal HIE in LMICs 66, 67. Although the standard treatment used a caffeine concentration of 20 mg/kg (with a follow daily dose of 5 mg/kg or 10 mg/kg depending on the severity 64), which is below the concentration used in this study (40 mg/kg), several studies using higher doses in premature babies did not show any neurodevelopmental damage over time 68, 69. Our PK study showed a mean peak of caffeine in plasma immediately after the first injection of 25.6 mg/mL. A pharmacokinetic study of 17 preterm infants with a postnatal age between 15 and 38 days (gestational ages: 26-33 weeks) and birth weights between 850 and 2130 g treated with 10 mg/kg (active base) following by 2.5 mg/kg (maintenance dose) of caffeine, showed peak plasma of caffeine concentrations ranging from 6 to 35 mg/L (mean = 17.8 mg/L), which were associated with a 40% reduction in apneic episodes, with a plasma caffeine half-life ranged from 20.4 to 99 h 70. While several studies point to variability in PK values depending on factors such as administration route, neonate weight, or gestational age, our PK values showed high bioavailability between the first 24 h, with plasma concentration in the range reported 71. Nevertheless, more studies in large animals should be conducted to determine the neuroprotective effects of a high dose of caffeine and the relationship between bioavailability and toxicity. Another important observation is that caffeine was administered prior to HI. It has been demonstrated that the administration of caffeine before insult reduces white matter diseases in a hypoxic-ischemic rat model 64, and improved amplitude-integrated EEG 72. Our study was planned to reflect an optimized clinical scenario in which caffeine could potentially be administered before the onset of hypoxia-ischemia (antenatally), particularly because caffeine can pass through the blood-brain barrier and the placenta.

This study had some limitations. Several publications point the importance of sex-dependent effects after HI 73-75. In our study we did not find any sex-differences between the treatment groups. However, it has to be acknowledged that the present work was not primarily designed and high enough powered to primarily target sex-differences. Nevertheless, previous studies of caffeine treatment following neonatal HI with similar animal numbers to our study, did also not find caffeine treatment to be sex dependent, but suggested a sex-dependent microglia response after caffeine treatment 76.

Regarding behavioral tests, we found that catwalk as a motor behavior test provides enough information regarding improvement after caffeine treatment; however, more tests that cover different aspects of cognitive development need to be tested, as well as the use of different ages to better cover cognitive development over time. For the long term study, MRI was used to determine the degree of insult in the animals. MRI has been used previously to pre-select animals for further experiments and has been determined to be a very important tool, particularly immediately after HI (24 h after HI) to further understand the benefits and limitations of further treatments, and to reduce the number of animals used for long-term studies (considering the large number of animals due to the high variability of this model). It has been demonstrated that despite little apparent difference at 24 h, it can also predict how the injury in the pup and the edema size evolves into infarction and large cavitation in the long term, and is a key tool to predict behavioral outcomes 26, 77. However, further research on the use of MRI as a prediction tool needs to be developed, including diffusion-weighted magnetic resonance imaging and extended analysis of the data, including the number of tracts, apparent diffusion coefficient, and fractional anisotropy 28.

Our results provide a mechanistic basis for the development of useful, reproducible, and potentially translational compounds by mechanistically understanding the effect of HI on the AMPK/mTOR pathway. We also provide new insights into the effects of caffeine on neonatal HI, with a deeper understanding of the mechanism of the caffeine effect and its use as a possible neuroprotective compound. These findings demonstrate that caffeine is a promising therapeutic agent against HIE, particularly in LMIC where TH is not easily accessible or does not show a promising outcome. This study showed that the beneficial effects of caffeine are mediated through AMPK/mTOR inhibition, and reduction of microgliosis. Exploring the role of AMPK/mTOR in a neonatal HI model provides an increased understanding of how this pathway contributes to pathology, and will be key for developing novel therapies in the future.

## Conclusion

Understanding how AMPK/mTOR activity is regulated following neonatal hypoxia-ischemia is not only important to better delineate the biological function of the pathway in translation but also to highlight potential therapeutic strategies for treating HIE and understanding the potential neuroprotective mechanisms. Investigations of drugs that target certain hubs of the complex cell death cascade are ongoing. We demonstrated that caffeine exerts a neuroprotective effect by reducing inflammation in the brain and regulating the AMPK/mTOR pathway immediately after HI in the most affected areas after HI: the cortex and hippocampus. Coupled with the fact that caffeine already has known biological efficacy and dose tolerability in human newborns, it may be rapidly and relatively cheaply repurposed for use as adjunct HIE therapy, particularly in LMIC.

## Supplementary Material

Supplementary figures and tables.
