# Supplementary material for: The Neuroprotective Effects of Caffeine in a Neonatal Hypoxia-Ischemia Model are Regulated through the AMPK/mTOR Pathway
Source: Int J Biol Sci. 2025 Jan 1;21(1):251–70. doi: 10.7150/ijbs.101087 (PMC11667826; doi:10.7150/ijbs.101087)
Supplement: Supplementary file 1 — Supplementary figures and tables. [file ijbsv21p0251s1.pdf]

Supplementary Figure 1: Sex correlation after treatment. **(A)** Sex correlation after the different dose treatments with caffeine 7-days after HI. Males: n=42 (HI/Vehicle), n=6 (caffeine 15 mg/kg – first dose before HI), n=12 (caffeine 20 mg/kg – first dose before HI), n=10 (caffeine 40 mg/kg – first dose before HI), n=7 (caffeine 40 mg/kg – first dose after HI). Females: n=35 (HI/Vehicle), n=2 (caffeine 15 mg/kg – first dose before HI), n=12 (caffeine 20 mg/kg – first dose before HI), n=13 (caffeine 40 mg/kg – first dose before HI), n=5 (caffeine 40 mg/kg – first dose after HI). **(B)** sex correlation (40 mg/Kg caffeine treatment) 60 days after HI. Males: n=5 (HI/Vehicle), n=5 (HI/Caffeine). Females: n=4 (HI/Vehicle), n=3 (HI/Caffeine). **(C)** sex correlation on the AMPK activation at 4 h and 24 h after HI in the cortex. **(D)** sex correlation on the mTOR activation at 4 h and 24 h after HI in the cortex. **(E)** sex correlation on the AMPK activation at 4 h and 24 h after HI in the hippocampus. **(F)** sex correlation on the mTOR activation at 4 h and 24 h after HI in the hippocampus. Male: blue dots and females: pink dots. Males 4h after HI: n=4 (sham), n=4 (HI/Vehicle), n=6 (HI/Caffeine). Males 24h after HI: n=4 (Sham), n=5 (HI/Vehicle), n=3 (HI/Caffeine). Females 4h after HI: n=4 (Sham), n=6 (HI/Vehicle), n=5 (HI/Caffeine). Females 24h after HI: n=4 (Sham), n=4 (HI/Vehicle), n=4 (HI/Caffeine). Nonparametric tests were performed using the Mann–Whitney U test with a 95% confidence interval with a *$p<0.05$ and ***$p<0.0001$. Data are expressed as the median (IQR).

Supplementary Figure 2: Brain damage on magnetic resonance imaging **(A)** P8 rats (24 h after HI) were scanned and score from to 1-4 from no damage to severe damage, based on the degree of edema and the area affected. **(B)** Representative pictures showing edema with different Bregma segments for samples with a score of 1-2, representing no or mild damage. The dotted blue line on the ipsilateral side shows the affected areas, mostly caudate putamen areas. **(C)** Representative pictures showing edema with different bregma segments for samples with a score of 3-4, representing moderate or severe damage. The dotted blue line on the ipsilateral side shows the affected areas, mostly the cortical and caudate putamen areas.

Supplementary Figure 3: Microglia and monocyte-derived macrophages protein activity following HI/Caffeine treatment. **(A)** Schematic image showing the dissection of the ipsilateral cortex and hippocampus. **(B)** Protein level was expressed as the ratio of Iba-1 in the cortex. Representative protein bands of a western blot for Iba-1 for the HI/Vehicle and HI/Caffeine at 4 and 24 h in the cortex. Actin indicates equal protein loading. **(C)** Protein level was expressed as the ratio of CX3CR1 (microglia marker)

in the cortex. Representative protein bands of a western blot for CX3CR1 for the HI/Vehicle and HI/Caffeine at 4 and 24 h in the cortex. Actin indicates equal protein loading. **(D)** Protein level was expressed as the ratio of CCR2 (monocyte-derived macrophages) in the cortex. Representative protein bands of a western blot for CCR2 for the HI/Vehicle and HI/Caffeine at 4 and 24 h in the cortex. Actin indicates equal protein loading. **(E)** Protein level was expressed as the ratio of Iba-1 in the hippocampus. Representative protein bands of a western blot for Iba-1 for the HI/Vehicle and HI/Caffeine at 4 and 24 h in the hippocampus. Actin indicates equal protein loading. **(F)** Protein level was expressed as the ratio of CX3CR1 (microglia marker) in the hippocampus. Representative protein bands of a western blot for CX3CR1 for the HI/Vehicle and HI/Caffeine at 4 and 24 h in the hippocampus. Actin indicates equal protein loading. **(G)** Protein level was expressed as the ratio of CCR2 (monocyte-derived macrophages) in the hippocampus. Representative protein bands of a western blot for CCR2 for the HI/Vehicle and HI/Caffeine at 4 and 24 h in the hippocampus. Actin indicates equal protein loading. HI/Vehicle n=9 (black dots), and HI/Caffeine n=9 (blue squares) for each time points. Nonparametric tests were performed using the Mann–Whitney U test with a 95% confidence interval with a *p<0.05 and ***p<0.0001. Data are expressed as the median (IQR). Image A made with Biorender.com.

Supplementary Figure 4: Astrocyte GFAP protein activity following HI/Caffeine treatment. **(A)** Schematic image showing the dissection of the ipsilateral cortex and hippocampus. **(B)** Protein level was expressed as the ratio of GFAP in the cortex. Representative protein bands of a western blot for GFAP for the HI/Vehicle and HI/Caffeine at 4 and 24 h in the cortex. Actin indicates equal protein loading. **(C)** Protein level was expressed as the ratio of GFAP in the hippocampus. Representative protein bands of a western blot for GFAP for the HI/Vehicle and HI/Caffeine at 4 and 24 h in the hippocampus. Actin indicates equal protein loading. HI/Vehicle n=9 (black dots), and HI/Caffeine n=9 (blue squares) for each time points. Nonparametric tests were performed using the Mann–Whitney U test with a 95% confidence interval with a *p<0.05. Data are expressed as the median (IQR). Image A made with Biorender.com.

Supplementary Figure 5: Neuronal NeuN protein activity following HI/Caffeine treatment. **(A)** Schematic image showing the dissection of the ipsilateral cortex and hippocampus. **(B)** Protein level was expressed

as the ratio of NeuN in the cortex. Representative protein bands of a western blot for NeuN for the HI/Vehicle and HI/Caffeine at 4 and 24 h in the cortex. Actin indicates equal protein loading. **(C)** Protein level was expressed as the ratio of NeuN in the hippocampus. Representative protein bands of a western blot for NeuN for the HI/Vehicle and HI/Caffeine at 4 and 24 h in the hippocampus. Actin indicates equal protein loading. HI/Vehicle n=9 (black dots), and HI/Caffeine n=9 (blue squares) for each time points. Nonparametric tests were performed using the Mann–Whitney U test with a 95% confidence interval with a *$p<0.05$. Data are expressed as the median (IQR). Image A made with Biorender.com.

Supplementary Figure 6: S6 and 4EBP-1 protein activity following HI treatment. **(A)** Protein level was expressed as the ratio of phosphorylated 4EBP-1 to total 4EBP-1 in the cortex. Representative protein bands of a western blot for 4EBP-1 for the sham, HI/Vehicle, HI/CC, and HI/Rap at 4 and 24 h at the cortex. Actin indicates equal protein loading. Arrow head point 4EBP-1 band analyzed. **(B)** Protein level was expressed as the ratio of phosphorylated S6 to total S6 in the cortex. Representative protein bands of a western blot for S6 for the sham, HI/Vehicle, HI/CC, and HI/Rap at 4 and 24 h at the cortex. Actin indicates equal protein loading. **(C)** Protein level was expressed as the ratio of phosphorylated 4EBP-1 to total 4EBP-1 in the hippocampus. Representative protein bands of a western blot for 4EBP-1 for the sham, HI/Vehicle, HI/CC, and HI/Rap at 4 and 24 h at the hippocampus. Actin indicates equal protein loading. Arrow head point 4EBP-1 band analyzed. **(D)** Protein level was expressed as the ratio of phosphorylated S6 to total S6 in the hippocampus. Representative protein bands of a western blot for S6 for the sham, HI/Vehicle, HI/CC, and HI/Rap at 4 and 24 h at the hippocampus. Actin indicates equal protein loading. Sham n=9 (white dot), HI n=9 (black dot), and HI/Rap (blue triangles) n=8 at both time points. HI/CC (pink triangles) n=7 at 4 h, n=5 at 24 h. One-way ANOVA followed by Tukey *post hoc* test for multiple comparison was used with *$p<0.05$ and ***$p<0.0001$. Data are expressed as the median (IQR).

Supplementary Figure 7: Caffeine effect on the translational factors 4EBP-1 and S6 protein activity following HI treatment. **(A)** Protein level was expressed as the ratio of phosphorylated 4EBP-1 to total 4EBP-1 in the cortex. Representative protein bands of a western blot for 4EBP-1 for the Sham, Sham/Caffeine, HI/Vehicle and HI/Caffeine groups in the cortex. Actin indicates equal protein loading. Arrow head point 4EBP-1 band analyzed. **(B)** Protein level was expressed as the ratio of phosphorylated

S6 to total S6 in the cortex. Representative protein bands of a western blot for S6 for the Sham, Sham/Caffeine, HI/Vehicle and HI/Caffeine groups in the cortex. Actin indicates equal protein loading. **(C)** Protein level was expressed as the ratio of phosphorylated 4EBP-1 to total 4EBP-1 in the hippocampus. Representative protein bands of a western blot for 4EBP-1 for the HI/Vehicle and HI/Caffeine groups in the hippocampus. Actin indicates equal protein loading. Arrow head point 4EBP-1 band analyzed. **(D)** Protein level was expressed as the ratio of phosphorylated S6 to total S6 in the hippocampus. Representative protein bands of a western blot for S6 for the Sham, Sham/Caffeine, HI/Vehicle and HI/Caffeine groups in the hippocampus. Actin indicates equal protein loading Sham n=9 (white dots), Sham/Caffeine n=7 (green triangles), HI/Vehicle n=9 (black dot), and HI/Caffeine n=9 (blue squares) for each time points. One-way ANOVA followed by Tukey *post hoc* test for multiple comparison was used with *p<0.05 and ***p<0.0001. Data are expressed as median (IQR).

Supplementary Fig. 1

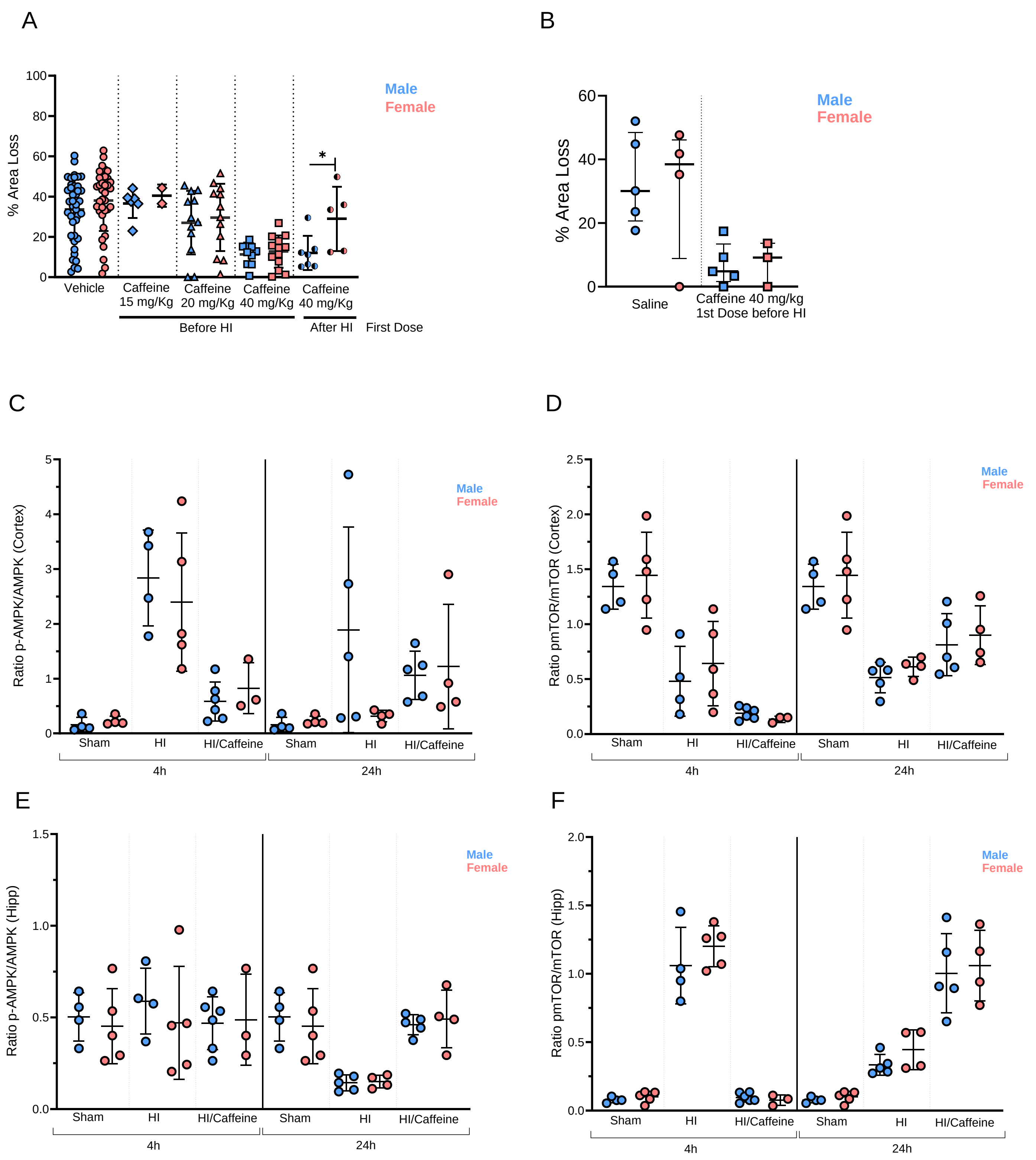

Supplementary Fig. 2

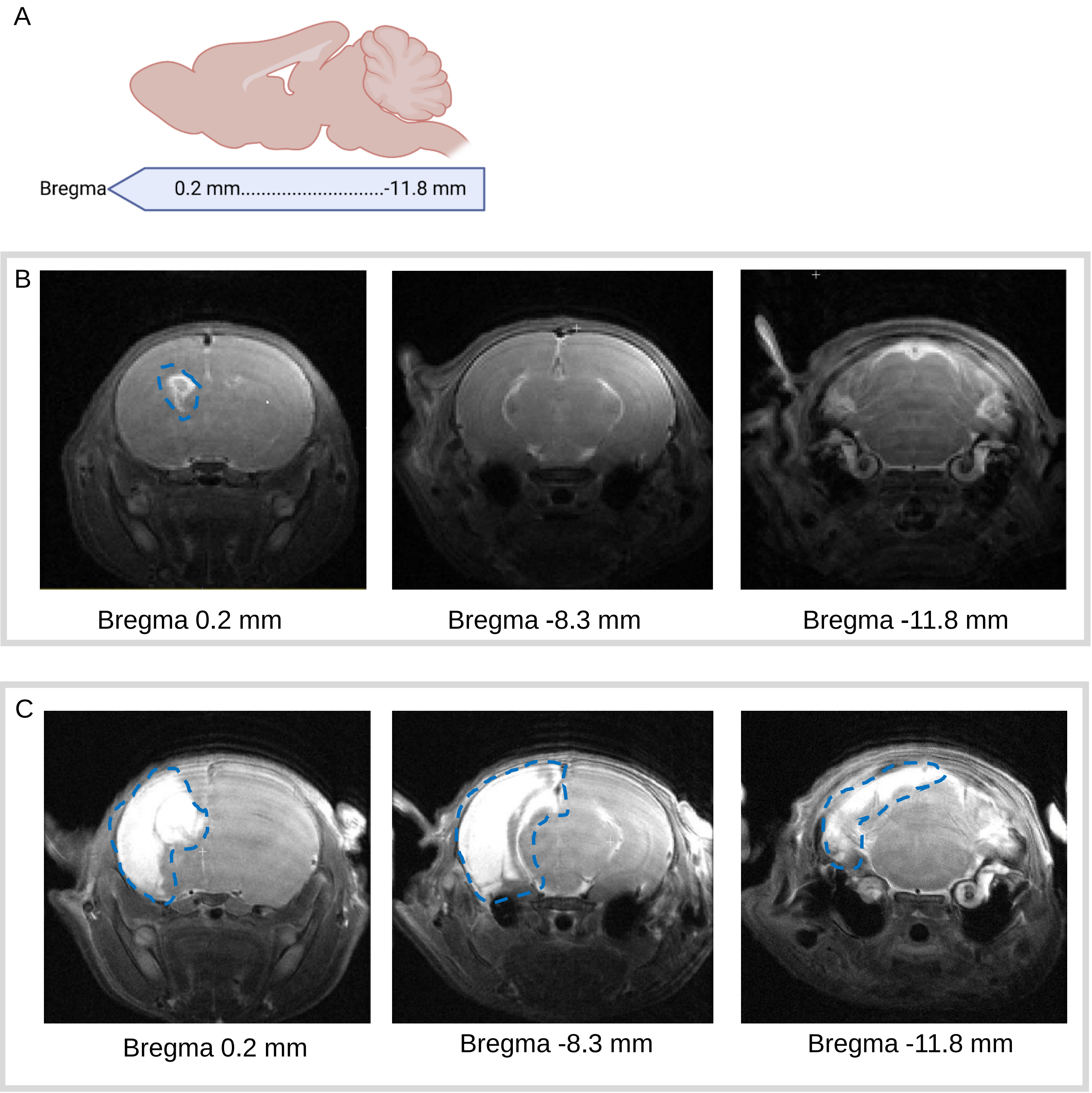

Supplementary Fig. 3

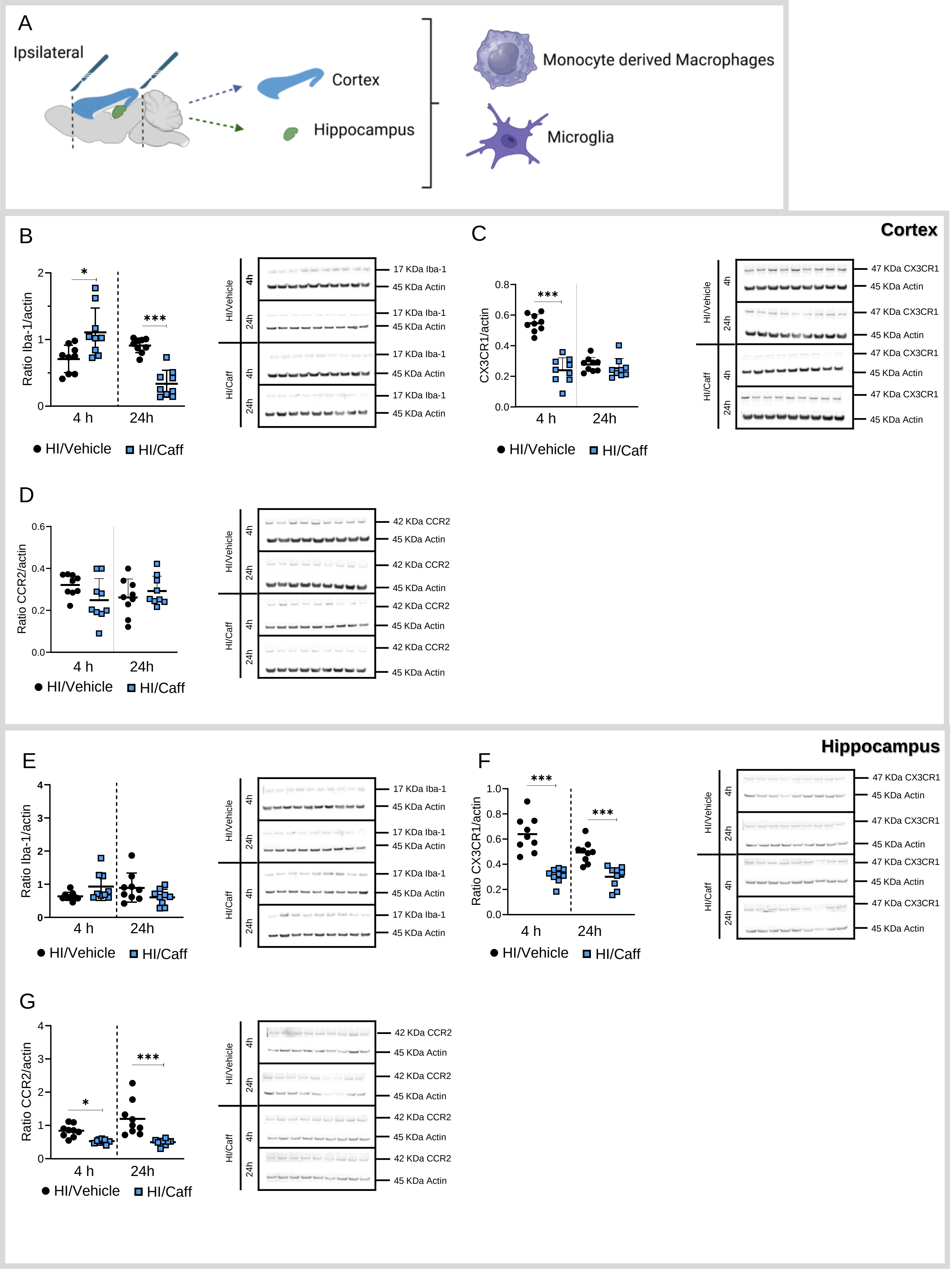

Supplementary Fig. 4

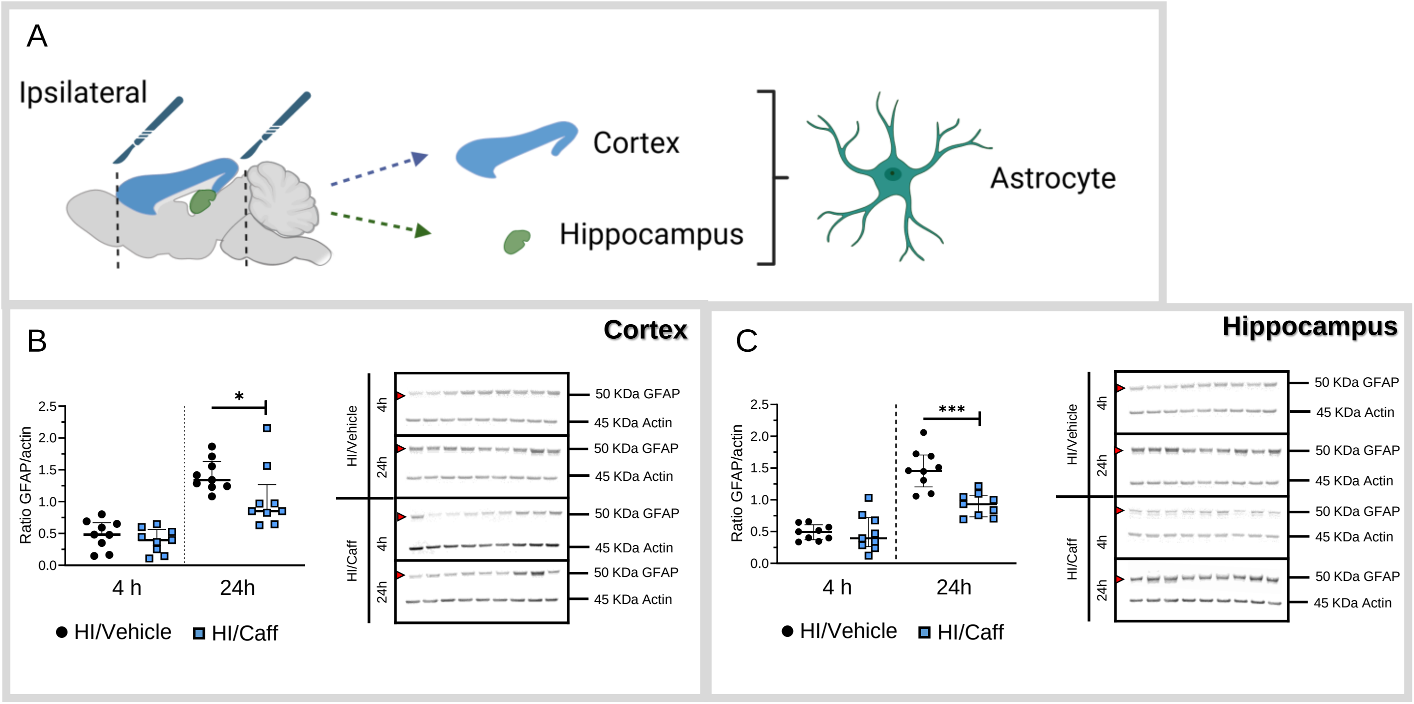

Supplementary Fig. 5

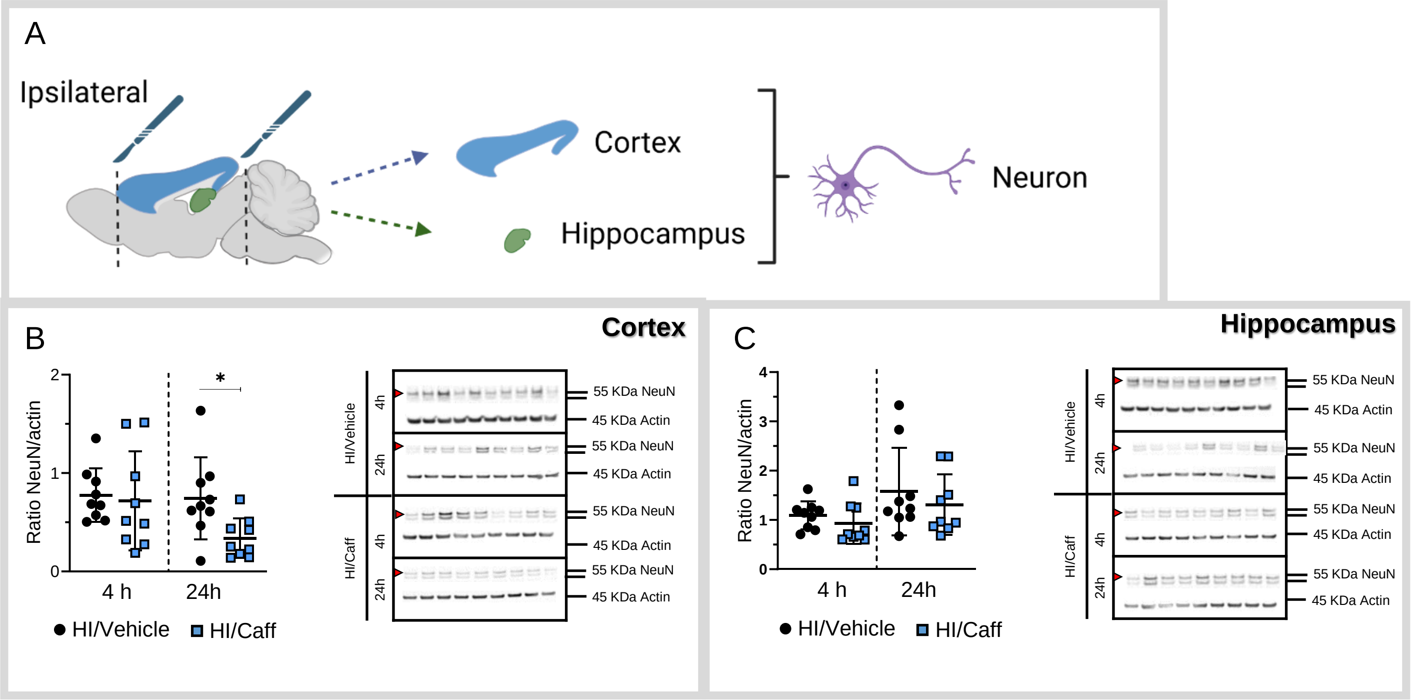

Supplementary Fig. 6

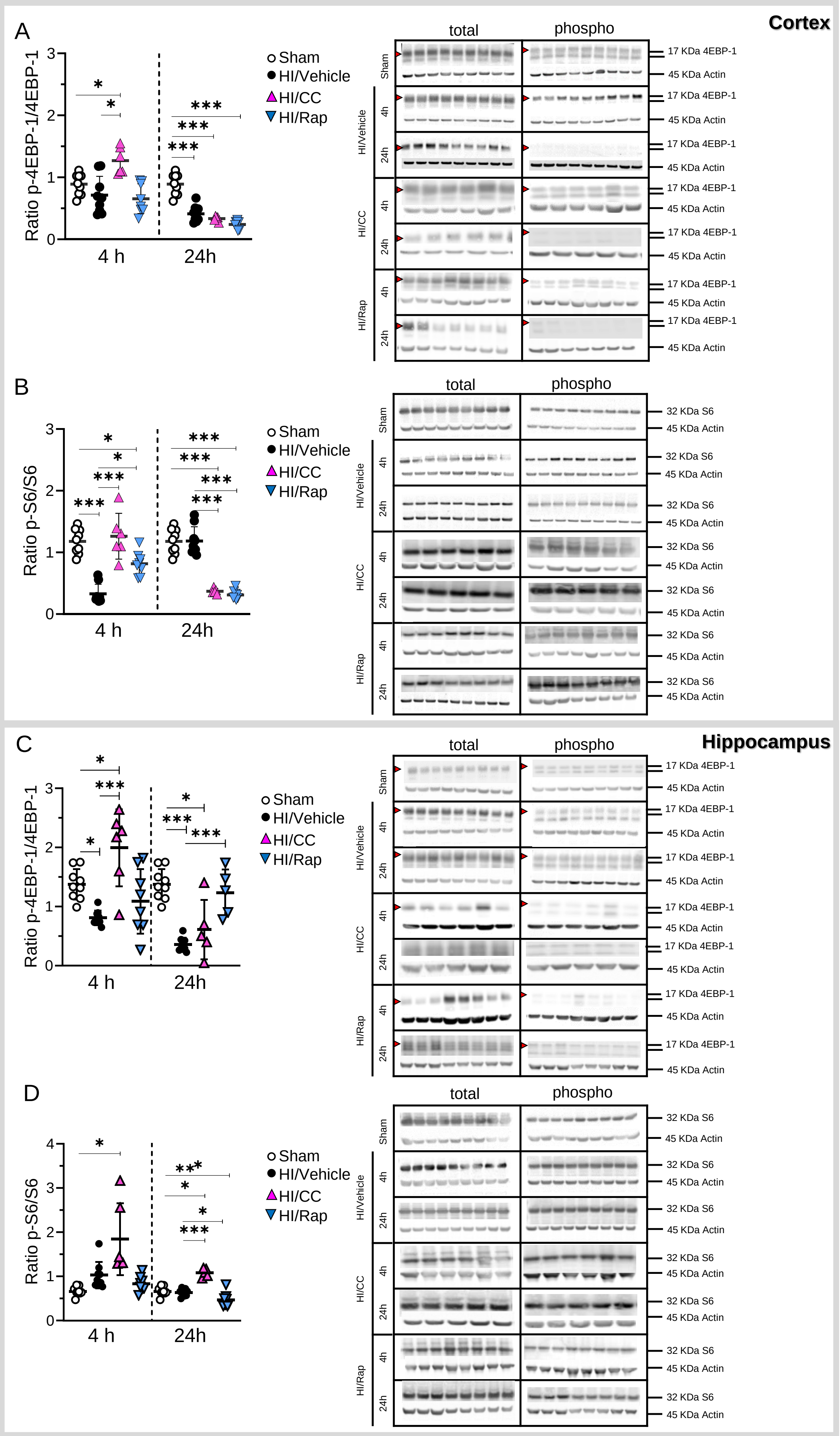

Supplementary Fig. 7

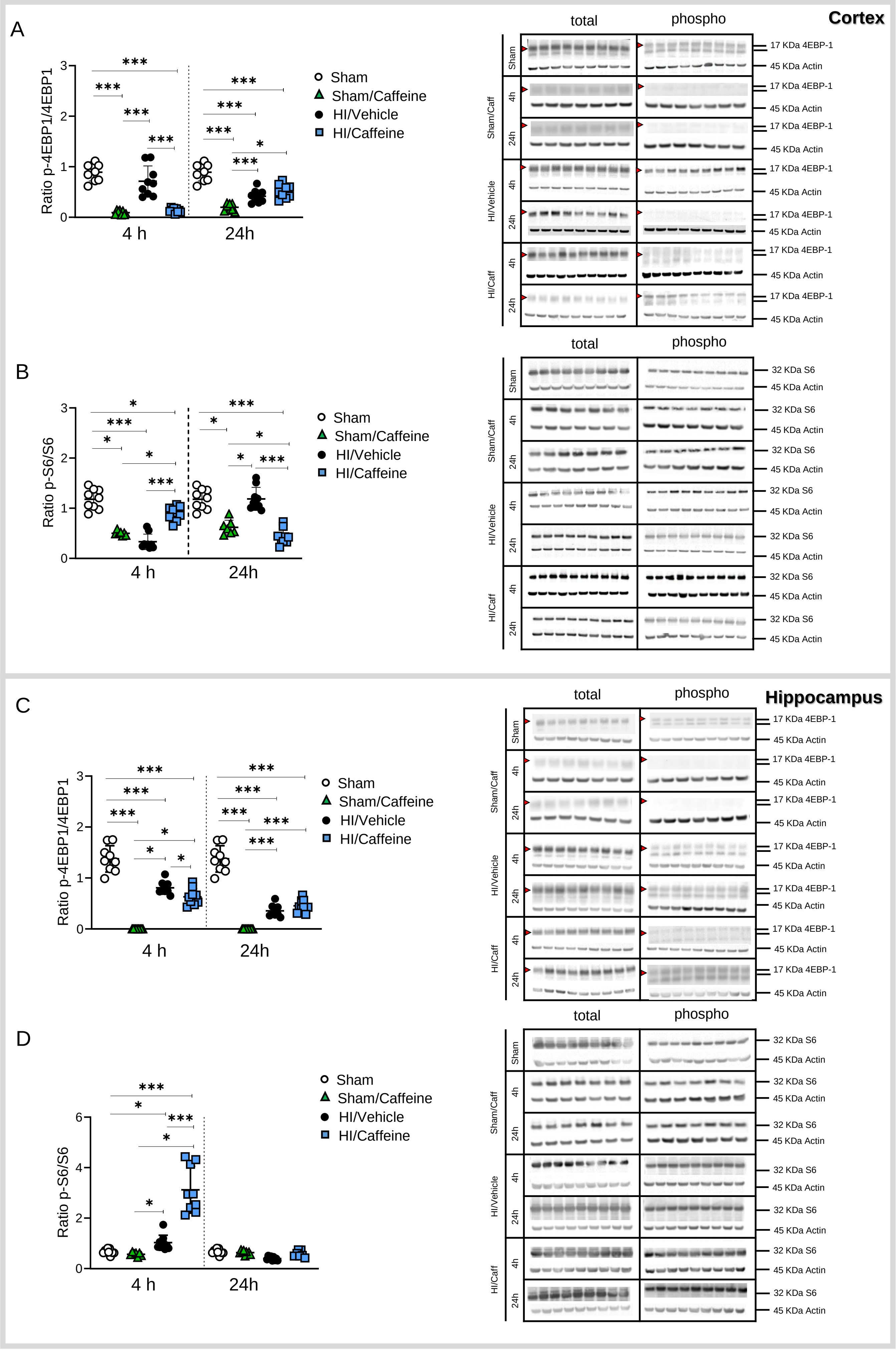

Supplementary Table1: Number of animals used for condition

Purpose	Treatment	Number	Mortality N	Exclude N
Western Blot 4/24h after HI	Sham	9	-	-
	Sham/Caffeine 40 mg/kg	7	-	-
	HI/Vehicle	18	-	-
	HI/Compound C	22	11*	-
	HI/Rapamycin	16	8*	1***
	HI/Caffeine 40 mg/kg	16	-	-
Pharmacokinetic 7-days after HI	HI/Vehicle	77$	-	-
	HI/Caffeine 15 mg/kg	8	-	-
	HI/Caffeine 20 mg/kg	20	-	-
	HI/Caffeine 40 mg/kg#	23$$	-	-
	HI/Caffeine 40 mg/kg##	12	-	-
	HI/Caffeine 120 mg/kg	14	14**	
Pharmacodynamic (0, 1, 2, 4, 8, 24 h after HI)	HI/Caffeine 40 mg/kg#	23	-	-
Behavior	HI/Vehicle	15	2	4
	HI/Caffeine 40 mg/kg#	15	1	6

* died during HI

** 5 animals died during HI and 9 animals died 48h after HI

*** brain severely damaged

first dose before HI

first dose after HI

$ 5 animals used for IHC

$$ 9 animals used for IHC

Supplementary Table 2: Antibody list

Technique	Cat. N°	Antibody	Supplier	Clone	Dilution WB	Dilution IHC	Host
Western Blot	2532	AMPK	Cell Signaling	-	1 in 500	-	rabbit
	2535	phospho AMPK Thr172	Cell Signaling	40H9	1 in 500	-	rabbit
	9644	4EBP-1	Cell Signaling	53H11	1 in 500	-	rabbit
	2855	phospho 4EBP-1 Thr37/46	Cell Signaling	236B4	1 in 500	-	rabbit
	2983	mTOR	Cell Signaling	7C10	1 in 500	-	rabbit
	2974	phospho mTOR Ser2448	Cell Signaling	-	1 in 500	-	rabbit
	2217	S6 ribosomal	Cell Signaling	5G10	1 in 500	-	rabbit
	5364	phospho S6 ribosomal Ser240/244	Cell Signaling	D68F8	1 in 500	-	rabbit
	16153-1-AP	CCR2	Proteintch	-	1 in 1000	-	rabbit
	13885-1-AP	CX3CR1	Proteintch	-	1 in 1000	-	rabbit
	A1978	β-actin	Sigma	AC-15	1 in 3000	-	mouse
	016-20001	Iba-1	Wako	-	1 in 500	-	rabbit
	35518	goat-anti-mouse IgG (H+L) DyLight™ 680 Conjugated	Invitrogen	-	1 in 3000	-	mouse
	SA535571	goat-anti-rabbit IgG (H+L) DyLight™ 800 Conjugated	Invitrogen	-	1 in 3000	-	rabbit
Western Blot & IHC	80788	GFAP	Cell Signaling	E4L7M	1 in 500	1 in 100	rabbit
	24307	NeuN	Cell Signaling	D4G4O	1 in 500	1 in 50	rabbit
IHC	019-19741	Iba-1	Wako	-	-	1 in 300	rabbit
	A11008	goat-anti-rabbit IgG (H+L) Cross-Adsorbed Alexa Fluor™ 488	Invitrogen	-	-	1 in 500	rabbit

Supplementary Table 3: Expression Levels of AMPK/mTOR

Protein	Condition	Time point (h)	Cortex		Hippocampus	
			Compared to Sham	Compared to HI/NT	Compared to Sham	Compared to HI/NT
AMPK	Sham/Caffeine	4		↓		↓
	HI/Vehicle		↑		↑	
	HI/CC			↓		↓
	HI/Caff			↓	↑	
	Sham/Caffeine	24				
	HI/Vehicle					
	HI/CC				↑	↑
	HI/Caff				↑	
mTOR	Sham/Caffeine	4	↓		↓	↓
	HI/Vehicle		↓			
	HI/CC			↑	↑	↑
	HI/Rap		↓		↓	
	HI/Caff		↓	↓	↓	↓
	Sham/Caffeine	24			↓	↓
	HI/Vehicle		↓		↓	
	HI/CC		↓	↓	↓	
	HI/Rap		↓	↓	↓	
	HI/Caff		↓	↑	↓	↑
4EBP-1	Sham/Caffeine	4	↓	↓	↓	↓
	HI/Vehicle				↓	
	HI/CC		↑	↑	↑	↑
	HI/Rap					
	HI/Caff		↓	↓	↓	↓
	Sham/Caffeine	24	↓	↓	↓	↓
	HI/Vehicle		↓		↓	
	HI/CC		↓		↓	
	HI/Rap		↓			↑
	HI/Caff		↓		↓	
S6	Sham/Caffeine	4	↓			↓
	HI/Vehicle		↓		↑	
	HI/CC			↑	↑	
	HI/Rap		↓	↑		
	HI/Caff		↓	↑	↑	↑
	Sham/Caffeine	24	↓	↓		
	HI/Vehicle					
	HI/CC		↓	↓	↑	↑
	HI/Rap		↓	↓	↓	↓
	HI/Caff		↓	↓		

↑/↓ significant
